# Spreading modes at slow-spreading ridges shifted by mantle heterogeneity of the asthenosphere

**DOI:** 10.1093/nsr/nwaf385

**Published:** 2025-09-11

**Authors:** Wei-Qi Zhang, Chuan-Zhou Liu, Min Xu, Boda Liu, C Johan Lissenberg, Henry J B Dick

**Affiliations:** State Key Laboratory of Submarine Geoscience, Second Institute of Oceanography, Ministry of Natural Resources, Hangzhou 310012, China; Laoshan Laboratory, Qingdao 266237, China; State Key Laboratory of Lithospheric and Environmental Coevolution, Institute of Geology and Geophysics, Chinese Academy of Sciences, Beijing 100029, China; College of Earth and Planetary Sciences, University of Chinese Academy of Sciences, Beijing 100049, China; College of Earth and Planetary Sciences, University of Chinese Academy of Sciences, Beijing 100049, China; State Key Laboratory of Tropical Oceanography, South China Sea Institute of Oceanology, Chinese Academy of Sciences, Guangzhou 510301, China; State Key Laboratory of Lithospheric and Environmental Coevolution, Institute of Geology and Geophysics, Chinese Academy of Sciences, Beijing 100029, China; School of Earth and Environmental Sciences, Cardiff University, Cardiff CF10 3AT, UK; State Key Laboratory of Marine Geology, Tongji University, Shanghai 200092, China

**Keywords:** mid-ocean ridge, abyssal peridotite, oceanic crust, mantle heterogeneity, detachment fault

## Abstract

Oceanic crusts at slow-spreading ridges are created either by symmetric spreading dominated by magmatic accretion or asymmetric spreading controlled by tectonic extension. Consecutive change in the spreading mode at the same ridge has been commonly attributed to variation in magma supply, but the mechanism controlling magma supply remains unclear. Here, we present geochemical analyses of peridotites and basalts from the Mid-Atlantic Ridge at 23°N, a region that has shifted from asymmetric spreading to symmetric spreading over the past 3.3 million years. Our results indicate that the asymmetric phase was characterized by a low magma flux, resulting from inherited ancient melt depletion in the asthenosphere. The subsequent increase in magma supply and shift to symmetric spreading corresponded with the arrival of more fertile mantle material. This work provides evidence of shifts between spreading modes driven by changing mantle compositions, highlighting the crucial role of asthenospheric heterogeneity in controlling the spreading modes at slow-spreading ridges.

## Teaser

Compositional heterogeneity of the asthenosphere modulates the seafloor spreading dynamics at slow-spreading ridges, exerting a primary control on the modes of mass and heat transfer between Earth’s interior and surface.

## INTRODUCTION

The global mid-ocean ridge (MOR) system, with a total length >65 000 km, is the first-order structure on Earth, along which oceanic crusts covering 70% of the Earth’s surface are created. The spreading modes of the MOR play a key role in controlling the mechanisms of crustal accretion and thus influencing the style and extent of mass and chemical exchange between Earth’s interior and surface [1,2]. Sufficient magma supply at fast-spreading ridges, such as the East Pacific Rise, results in symmetric spreading [3]. Nevertheless, two types of spreading modes, i.e. symmetric and asymmetric, co-exist at the slow-spreading ridges [3,4], such as the Mid-Atlantic Ridge (MAR). Symmetric spreading is dominated by magmatic accretion under a high magma supply, which is typically associated with normal crustal thickness (5–6 km) and symmetric high-angle normal faulting [5,6]. In contrast, asymmetric spreading is accommodated by tectonic extension under a reduced magma supply [3,7], resulting in the development of detachment faults that exhume plutonic rocks as oceanic core complexes (OCCs). It has been estimated that each spreading mode occupies half of the MAR and exerts different influences on seafloor morphology as well as lithospheric alteration (e.g. hydration and carbonatization) [4,8].

In particular, a temporal shift of spreading modes has been observed for some ocean ridges [9,10]. Although such a shift has been consensually ascribed to the variation in magma supply [10], the mechanism for the secular variation in magma supply at the same ridge remains unclear. Different factors, including mantle composition [11–14], upwelling dynamics [15–17], and temperatures [18], have been proposed to explain the change in magma supply, among which the mantle composition has been underestimated. This is because the asthenosphere underlying the ocean ridges has been commonly regarded as homogeneous in composition, based on studies of mid-ocean ridge basalts (MORBs) [19,20]. However, studies on abyssal peridotites reveal the existence of refractory mantle within the asthenosphere that were previously melt-depleted [14,21,22]. Entrainment of refractory mantle domains reduces the fertility of the asthenosphere, which would doubtlessly lower magma productivity [13,14,23] and facilitate the nucleation of detachment faulting [5,7,15,24,25]. Conversely, melting of the asthenosphere with more fertile lithologies can enhance magmatism [26], possibly terminating earlier detachments. Such an inference is supported by the occurrence of refractory mantle in some OCCs [11,27]. However, a systematic study on the causal relationship between refractory mantle domains and the shift in spreading mode is still scarce.

**Figure 1.**
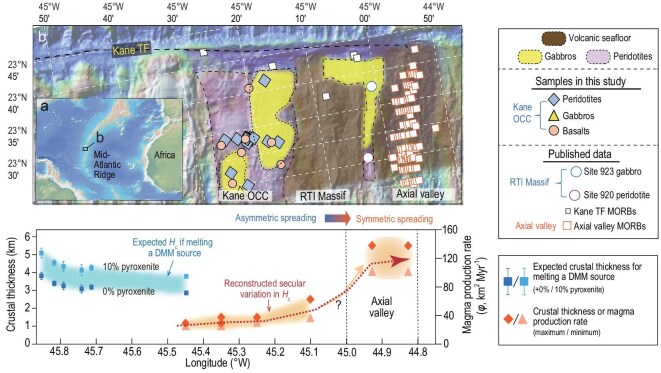
Regional geology and temporal variations in magma supply of MAR 23°N. (a) Bathymetric map of the Mid-Atlantic Ridge. (b) Bathymetric map of MAR 23°N from GeoMapApp (www.geomapapp.org), with distribution of basement lithologies [24,28]. (c) The reconstructed and expected original crustal thickness and magma production rate (φ). The moving-average peridotite melting degrees at different locations were utilized to compute the expected crustal thickness assuming melting a fertile DMM source under a Tp of 1350°C in the Melt-PX model [45] as shown in panel c.

The MAR south of the Kane Transform Fault (Kane TF) at 23°N provides an ideal location to test the role that asthenosphere heterogeneity played in shifting the spreading mode, as it has undergone a switch from asymmetric to symmetric spreading over the past 3.3 million years (Myr) [5,28]. During this period, three geological units have been produced (Fig. 1a, b): the Kane OCC (3.3–2.1 Ma), the Ridge-Transform Intersection (RTI) Massif (2.0–0.4 Ma), and the axial valley (0.4 Ma to present) [5,28]. Both the Kane OCC and RTI Massif were generated by detachment faulting under low magma supply. In contrast, the current axial valley at MAR 23°N exhibits a symmetric spreading under a high magma flux, as exemplified by a thick crust [5,24,28], an active melt lens [5], and a basalt-hosted hydrothermal field [29]. Such a contrast, i.e. long-lived asymmetric spreading from >3.3 Ma to 0.4 Ma followed by a switch to symmetric spreading with robust magmatism, is also supported by seismic tomography [5,24], which reveals a thick crust below the axial valley but thin crusts for both the Kane OCC and RTI Massif (Fig. S1a, b).

Plentiful oceanic rocks occur in these three units, in particular mantle peridotites exhumed in both the Kane OCC and RTI Massif, providing opportunities to reveal the temporal variations in compositions of the asthenosphere. Here, we present a multidisciplinary investigation of asthenosphere heterogeneity and crustal production at MAR 23°N, with an aim to highlight the crucial role of mantle heterogeneity in modulating seafloor spreading mode and crustal accretion at slow-spreading ridges. Detailed regional geology and analytical methods are provided in Notes S1 and S2, respectively.

## RESULTS

### Elevated magma flux during the transition in spreading modes

To constrain the secular variation in magma supply at MAR 23°N, we reconstruct the original crust thickness (*H*_c_, the effective initial thickness of magmas accreted on the ocean ridge) for both the asymmetric terranes and the axial valley by leveraging previous seismic results [5,24]. For asymmetric terranes, namely, the Kane OCC and RTI Massif, we integrated magma volumes distributed in both the footwall as gabbros and the hanging wall as extrusive rocks to calculate the total magma flux (φ), defined as the magma volume accreted per ridge length and per time, following the methodology of ref. [30]. We then converted φ into *H*_c_, assuming a full spreading rate of 25 mm yr^−1^. This approach enables a direct comparison of magma flux and *H*_c_ through time at 23°N. Details for the reconstructions of φ and *H*_c_ are provided in Materials and Methods. High-resolution seismic tomography suggests that the western part of the Kane OCC (WKO) contains fewer gabbros than the eastern part (EKO; Fig. S1c). Therefore, the magma flux for WKO and EKO were reconstructed separately.

Our reconstruction results reveal low magma fluxes for both WKO (φ = 26–29 km^2^ Myr^−1^, *H*_c_ = 1.0–1.2 km) and EKO (φ = 31–38 km^2^ Myr^−1^, *H*_c_ = 1.2–1.5 km). Moreover, the RTI Massif is reconstructed to have a low φ of 38–63 km^2^ Myr^−1^ and low *H*_c_ of 1.5–2.5 km. In contrast, a thick crust with *H*_c_ of 4.0–5.5 km is estimated to occur at the current axial valley, corresponding to a high magma flux of 100–138 km^2^ Myr^−1^. These results support an increase in magma supply during the transition in spreading modes at MAR 23°N, i.e. from 26–63 km^2^ Myr^−1^ during asymmetric spreading to 100–138 km^2^ Myr^−1^ at the axial valley (Fig. 1c).

### Mantle peridotites from the Kane OCC record ancient melt depletion

Thirty residual peridotites from the Kane OCC have been analyzed, including whole-rock and mineral geochemistry, highly siderophile elements (HSEs) and Re–Os isotopes. The Kane peridotites have been pervasively altered as indicated by their high loss on ignition (LOI) values (>10 wt%), which have negligible effects on their bulk Al_2_O_3_ contents, HSE abundances, and Os isotopes (Fig. S2; Note S3). The Kane peridotites contain 40–44 wt.% MgO and 0.7–2.1 wt.% Al_2_O_3_, typical of residual abyssal peridotites (Fig. S3a). Their spinel contains <0.1 wt.% TiO_2_ and have a Cr# [= 100 × Cr/(Cr + Al)] of 27–42 (Fig. S3b), yielding melting extents of 10%–16% from depleted MORB mantle (DMM) based on the empirical relationship from ref. [31]. Cpx (clinopyroxene) and Opx (orthopyroxene) in the Kane peridotites show light rare earth element depletion relative to heavy rare earth elements (Fig. S3c, d). Light rare earth element (LREE) compositions in abyssal peridotite pyroxenes are influenced by incomplete melt extraction and refertilization [32,33]. We therefore employed heavy rare earth elements in pyroxenes, based on a DMM fractional melting model [34], to infer melt depletion extents of 10%–16%, comparable to spinel Cr# estimates (Fig. S3c, d).

**Figure 2.**
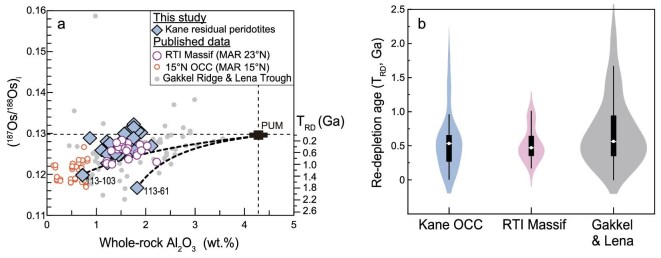
Re–Os isotopic data for the Kane OCC peridotites. (a) Initial ^187^Os/^188^Os versus 100% anhydrous whole-rock Al_2_O_3_. The binary mixing trends between PUM and two ancient peridotites (113–61, 113–103) are plotted for comparison. (b) Violins show the density of T_RD_ values and box and whisker symbols show interquartile range and median T_RD_.

The Kane peridotites exhibit flat patterns for Os, Ir, Ru, and Pt but variable and depleted Pd and Re compositions (Fig. S4). They have unradiogenic ^187^Os/^188^Os of 0.117–0.131 and correspondingly Re-depletion ages [T_RD_, relative to primitive upper mantle (PUM)] [35] as old as 1800 Ma (with a peak at 500 Ma; Fig. 2), which are much older than the age of the oceanic crust (<3 Ma).

### Variations in MORB compositions during the transition in spreading modes

Geochemical data of basalts erupted during asymmetric and symmetric spreading in the studied area (45.5–44.8°W, 23.2–23.6°N) were compiled from the PetDB database to assess temporal variations in their source compositions. These data are grouped by location (Fig. 1b): the Kane OCC (*n* = 20), the Kane TF (*n* = 38), and the axial valley (*n* = 152). In addition, we have supplemented new geochemical data of 7 gabbros and 12 basalts from the Kane OCC.

The Kane OCC basalts erupted off-axis in response to high-angle normal faulting during the cessation stage of detachment at 2 Ma [36], which have a median (La/Sm)_N_ of 0.58 (CI-chondrite-normalized value, ref. [37]). Melts in equilibrium with the Kane OCC gabbros that formed on-axis have a median (La/Sm)_N_ of 0.56, which are comparable to the Kane OCC basalts but lower than the axial valley MORB (0.65, Fig. S5a). Considering the similarity in Nd isotopes between the Kane OCC gabbros and basalts, we suggest that they originated from similar mantle sources. Thus, the geochemistry of the Kane OCC basalts reflects the source characteristics during asymmetric spreading.

**Figure 3.**
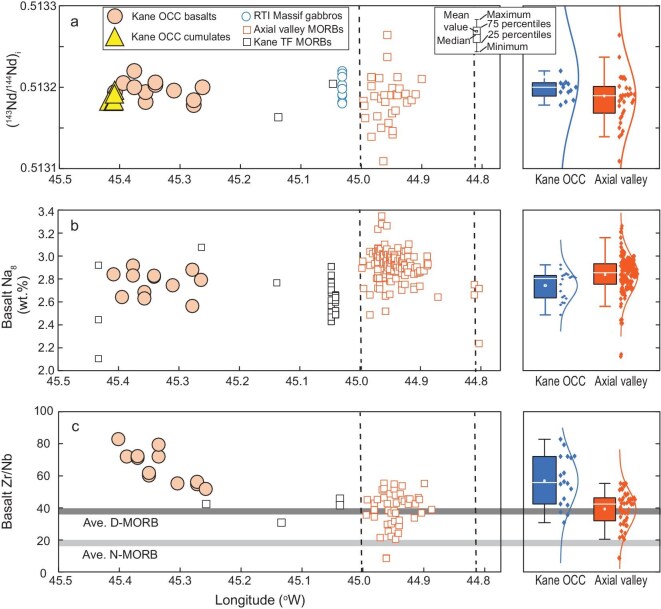
Cross-axis basalt geochemical variations. (a–c) Basalt initial ^143^Nd/^144^Nd, Na_8_, and Zr/Nb. Data are also displayed as box plots and kernel density distributions of basalts erupted during asymmetric and symmetric spreading stages. The ends of the boxes show the upper and lower quartiles, and the ends of the whiskers show the 2.5 and 97.5 percentiles.

The Kane OCC basalts have initial ^143^Nd/^144^Nd ratios of 0.51319 ± 0.00001, indistinguishable from the RTI Massif gabbros (0.51320 ± 0.00001, Fig. 3a, Fig. S5b). Compared to the axial valley basalts, the Kane OCC basalts display lower Na_8_ (Na_2_O contents corrected to a MgO value of 8 wt.%, refs [38,39]; Na_8_ = 2.7 ± 0.1), lower (La/Sm)_N_, higher Zr/Nb, and more depleted Nd isotopes (Fig. 3, Fig. S5a, b). Of note, Na_8_ and (La/Sm)_N_ in MORB are not simple proxies for the degree of melting but instead record the combined influences of source composition and melting extent [40,41]. A positive correlation between Zr/Nb and ^143^Nd/^144^Nd (*R*^2^ = 0.3) exists for all MAR 23°N basalts. In trace element diagrams, the Kane OCC basalts show a negative Nb anomaly, whereas the axial valley basalts display no Nb anomaly (Fig. S5c, d). In sum, the MORB erupted during asymmetric spreading are geochemically more depleted than those formed during the symmetric phase (Fig. 3, Fig. S5).

## DISCUSSION

### A link between low magma flux and ancient mantle during asymmetric spreading

**Figure 4.**
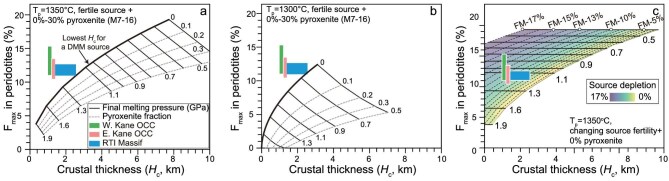
Melt-PX modeling. (a, b) Melting of a fertile DMM-like lherzolitic source plus minor pyroxenite (M7-16) under Tp = 1350°C (a) and Tp = 1300°C (b). (c) Melting of mantle source with variable prior melt depletion under Tp = 1350°C. F_max_ (%) in the three panels denotes the maximum extent of partial melting recorded in the abyssal peridotites, i.e. melting extents at the final melting depths. WKO = western part of the Kane OCC, EKO = eastern part of the Kane OCC, RTI = Ridge-Transform Intersection Massif, FM = fertile mantle. The model results indicate that the low magma flux but relatively refractory peridotite compositions cannot be explained by melting a fertile DMM source (a, b) but are consistent with melting a refractory mantle source with 8% prior melt depletion (c).

Crustal thickness (*H*_c_) during asymmetric spreading at MAR 23°N has been reconstructed as 1.5–2.5 km, which is substantially lower than the 5−7 km expected for symmetric slow-spreading ridges at a full spreading rate of 25 mm yr^−1^ [16]. This suggests a low magma supply at MAR 23°N during this period, which might be attributed to various factors, including spreading rate, mantle potential temperature (*T*_p_), and mantle compositions. First of all, the spreading rate effect can be ruled out, as the full spreading rate at MAR 23°N has remained unchanged over the past 3 Myr [5]. Second, the low magma supply is unlikely to be ascribed to a low *T*_p_, as the seismically inferred *T*_p_ beneath MAR 23°N is comparable to that in plume-unaffected segments, with no evidence for an underlying cold mantle (Fig. S6). We have conducted the experimentally-parameterized algorithm Melt-PX simulations (see Methods for details), showing that, at a final melting pressure of 0.9 GPa [12], reducing *T*_p_ from 1350°C to 1300°C decreases *H*_c_ from 4.0–4.5 km to 1.5–2.0 km (Fig. 4a, b). However, at *T*_p_ of 1300°C, the computed peridotite melting degree (F = 8%) is far too low to match the melting extents of 10%–16% estimated for the Kane OCC peridotites (Fig. S3b). Moreover, a 50°C thermal anomaly for a 30-km mantle blob would equilibrate within 0.1 Myr [11], making sustained low Tp from 3.3 Ma to 0.4 Ma untenable at MAR 23°N. It has been suggested that transform faults may raise the final melting pressure, thereby reducing magmatism in adjacent oceanic ridges [42]. However, to reproduce the low *H*_c_ (1.5–2.0 km) at the Kane OCC, the final melting pressure must reach 1.6 GPa, yielding a peridotite melting extent of <6%—far lower than the 10%–16% estimated for Kane peridotites (Fig. S3a). Moreover, it has been suggested that the ‘transform effect’ extends only 10 km from the Kane TF [43]. Therefore, the ‘transform effect’ cannot account for the low magma supply during asymmetric spreading at MAR 23°N [42].

Here we propose that the low magma flux during asymmetric spreading at MAR 23°N was due to entrainment of refractory mantle lithologies within the asthenosphere, which have inherited ancient melt depletion and thus have refractory compositions [11,14,23]. Entry of such refractory mantle into the melting zone beneath an ocean ridge can suppress adiabatic melting, thereby reducing magma productivity [14]. Such an interpretation is supported by the Re–Os isotopes of residual peridotites from both the Kane OCC and RTI Massif (Fig. 2). These peridotites exhibit unradiogenic ^187^Os/^188^Os and ancient Re-depletion ages of 0.4–1.8 Ga, with a median age of 500 Ma (Fig. 2). A similar explanation has been applied to the thin oceanic crusts at other ultraslow-spreading ridges (e.g. Gakkel Ridge and Lena Trough) [40,44], at which occurrence of ancient refractory mantle domains in the asthenosphere is inferred by unradiogenic Os isotopes of abyssal peridotites (Fig. 2).

To further test the role of refractory mantle in suppressing magmatism, we explored the relationship between crustal thickness and melting degree (Fig. 4), using the Melt-PX [20,45]. At slow-spreading ridges such as MAR, a time gap of 2.2 Myr has been documented between basaltic crust accretion and the exhumation of the residual mantle [46], corresponding to a 55-km distance along the spreading direction at MAR 23°N (Fig. 1c). These observations indicate that the peridotites from the Kane OCC and RTI Massif are associated with basaltic crust formed between 5.5 and 2.6 Ma—a period marked by magma-starved, asymmetric spreading [36]. Spinel Cr# was employed as a proxy for melting extent, revealing melting degrees of 10%–16% for peridotites from both the Kane OCC and RTI Massif (Fig. S7a). We have modeled melting of a fertile DMM source containing 0%–10% pyroxenite, using the average melting degrees inferred from the peridotites (Fig. S7b). Under such conditions, the expected crustal thickness at MAR 23°N between 45.7°W and 45.3°W would be 3.5–5.0 km, which are much thicker than the reconstructed thickness of the Kane OCC (1.0–1.5 km; Figs. 1c and 4a). To reconcile this discrepancy, we re-ran our Melt-PX simulations using a source that had already experienced 0%–17% melting from a fertile DMM [20]; these simulations reconcile the thin crust with a refractory mantle source that underwent ∼8% prior melting (Fig. 4c). Notably, Earth’s asthenosphere has been progressively melt-extracted and insufficiently homogenized by convection over geological time [44,47,48], implying that the ancient melt extraction recorded in the Kane peridotites requires a source less depleted than present-day DMM [19]. We interpret the ∼8% prior melt extraction for the Kane OCC mantle source as a conservative minimum estimate.

**Figure 5.**
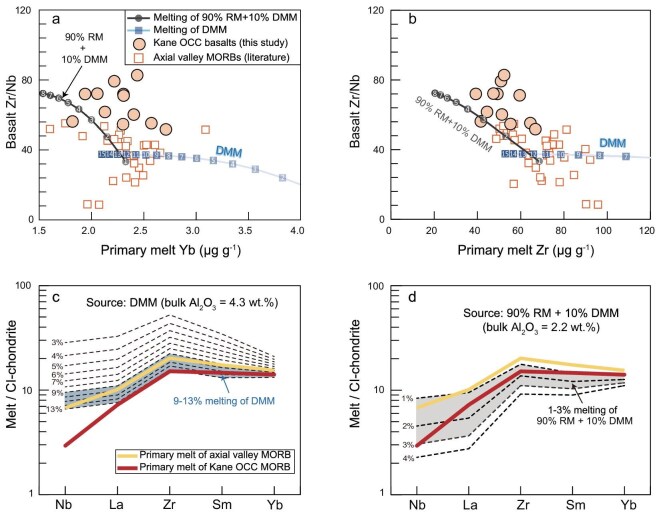
Geochemical modeling of basalt trace element systematics. (a, b) Basalt Zr/Nb versus primary melt Yb and Zr contents. White numbers in each modeling curve denote the melting degree (%). (c, d) Geochemical modeling of dynamic mantle melting reproducing the Nb, Zr, and REE compositions of primary melts of the Kane OCC and axial valley MORB. Details of modeling are provided in Note S4. RM = refractory mantle, DMM = depleted MORB mantle.

On the other hand, the geochemistry of the Kane OCC basalts is consistent with partial melting of such a source containing refractory mantle. The geochemical model shows that the aggregated partial melts from DMM [19] exhibit low Zr/Nb ratios of <40 (Fig. 5a–c; Note S4), which are exemplified by values of both N-MORB (19 ± 11) and D-MORB (39 ± 20, Fig. 3c). However, the Kane OCC basalts have higher Zr/Nb ratios of 52 to 79 (Fig. 3c), which cannot be explained by melting a fertile DMM source but imply the involvement of refractory mantle [49]. Furthermore, the primary melts of the Kane OCC are too Nb-depleted to be explained by DMM melting [see Notes S4 and S5 for details on mantle melting modeling (Fig. S8a) and reconstructing primary melts (Fig. S9), respectively]. Employing the method outlined in ref. [50], we reconstructed the bulk compositions of refractory mantle formed by 1%–24% melting within a triangular melting region (Fig. S8a). The Nb, Zr, and REE systematics of the primary melts of the Kane OCC basalts can be reproduced by low-degree melting (<4%) of a mixed source of 90% refractory mantle and 10% DMM (Fig. 5). This mixture yields bulk compositions (Al_2_O_3_ of 2.2 wt.% and Yb of 0.2 μg g^−1^) comparable to a 7%–8% melting residue of DMM (Fig. S8b), consistent with our Melt-PX simulations (Fig. S7d). These results indicate that ancient refractory mantle domains as old as 1.8 Ga were involved in the asthenosphere, causing low magma supply during the asymmetric spreading of MAR 23°N.

### Elevated source fertility during the transition in spreading modes

Substantial increase in magma flux has been inferred during the transition in spreading modes at 23°N on the MAR (Fig. 1c), consistent with patterns of magma flux variation observed during spreading-mode transitions at other slow-spreading ridges [10]. Here, various mechanisms related to the rapid increase in magma supply at MAR 23°N are evaluated, including (i) elevated *T*_p_ [18], (ii) enhanced buoyant mantle flow [51], (iii) enhanced magma focusing [51,52], and (iv) increase in source fertility [11–14]. First, as discussed above, rapid fluctuation in mantle temperature over the past 3.3 Myr at MAR 23°N is not supported by thermal calculations [11] nor the seismically-inferred *T*_p_ along the MAR [53] (Fig. S6). Enhanced buoyant mantle flow rarely induces remarkable crustal thickness variations at slow-spreading ridges [16,52]. Enhanced magma focusing redistributes melt within a segment at slow-spreading ridges, possibly thickening the local oceanic crust near the segment center [52]. However, magma focusing does not alter the segment-averaged melt volume. The average magma flux for the ridge segment between 21°N and 24°N on the MAR increased, with the magmatically active zone propagating northward over the past 5 Myr [10]. Therefore, the enhanced magmatism at MAR 23°N likely corresponded to the elevated magma flux in the 21°N–24°N segment [10]. Furthermore, it is challenging to explain enhanced melt focusing over a short period (<0.4 Myr) without considering changes in source characteristics. The upwelling of fertile lithologies can induce more melts from deep melting, enhancing magma focusing [10,26]. Hence, the shifts between spreading modes at MAR 23°N are not linked to fluctuation in mantle temperature, enhanced mantle upwelling, or enhanced magma focusing.

The final possibility for the substantial increase in magma flux during the shifts between spreading modes at MAR 23°N is a change in source composition and lithology [11–14]. Following the transition to symmetric spreading at MAR 23°N, the basalts became more geochemically enriched, exhibiting an increase in Na_8_, and enrichments in highly incompatible elements such as lower Zr/Nb ratios reflecting relative Nb enrichment (Fig. 3) and elevated (La/Sm)_N_ (Fig. S5a). This geochemical transition can result from either a decreasing extent of melting from a common source or the involvement of fertile mantle lithologies. The first hypothesis suggests that the more enriched basalt compositions in the axial valley reflect lower mantle melting extent compared to those of the Kane OCC, which contradicts the higher magma flux in the axial valley (Fig. 1c). Our mantle melting modeling suggests that the primary melts of the axial valley MORB are consistent with 9%–13% melting of the DMM-like source (Fig. 5c), corresponding to the observed thick axial crusts (Fig. 1c). In contrast, the low magma flux and depleted primary melt compositions for the Kane OCC are consistent with low-degree melting of a refractory mantle lithology (Al_2_O_3_ = 2.3 wt.%) that was less fertile than DMM (Al_2_O_3_ = 4.3 wt.%; Fig. 5d). Hence, rather than reflecting declining melting degrees, the basalt geochemical transition at MAR 23°N indicates that the source had become more fertile and enriched during the transition in spreading mode. Elevated source fertility below the axial valley likely results from a reduction in ancient depleted mantle proportion and/or the incorporation of more fertile lithologies [54]. The latter hypothesis is supported by the increased isotopic and geochemical variability during the spreading-mode transition (Fig. 3) and by local enriched MORB occurrences on axis at MAR 23°N [54]. An overall fertile but heterogenous mantle source comprising both depleted and enriched lithologies is thus likely present underneath the axial valley at MAR 23°N. Given uncertainties in the exact lithological heterogeneity of the axial valley mantle, we focus on mean source fertility when comparing crustal generation across spreading phases.

**Figure 6.**
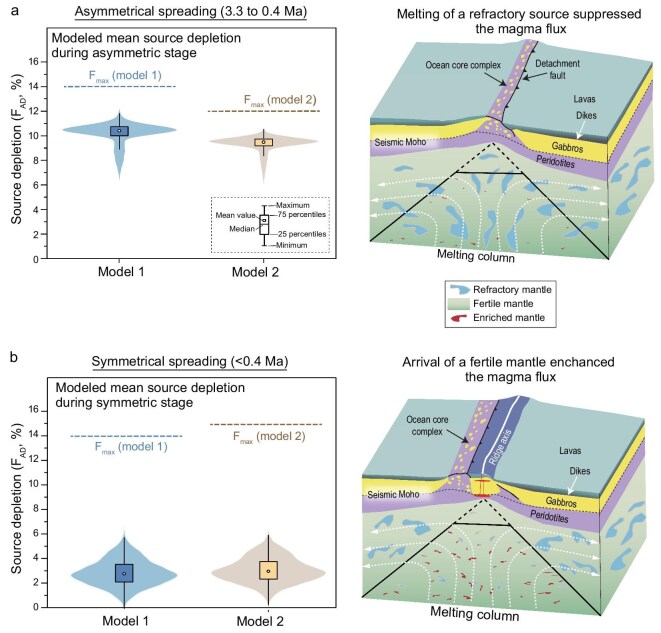
Shift in spreading modes below MAR 23°N over the past 3.3 Myr linked to changing source depletion (F_AD_). Source depletion (F_AD_) is defined as the prior melt depletion relative to fertile DMM [19]. Violins show the density of F_AD_ values and box and whisker symbols show interquartile range and median F_AD_. Both the modeled F_AD_ and F_max_ are plotted. Modeled F_AD_ for the mantle source of the asymmetric spreading phase (a) and symmetric spreading phase (b) of MAR 23°N at 3.3 to 0.4 Ma, with cartoons showing the possible source characteristics for each period. The forward mantle melting model considers either constant *H*_f_ [Model 1, *H*_f_ = 19.5 km, corresponding to a maximum melting degree (F_max_) of 13.5%] or varying *H*_f_ [Model 2, *H*_f_ = 15–24 km, F_max_ = 12%–15%]. White dashed arrays denote the inferred trajectory of mantle flow.

To assess the feasibility of reproducing the crustal thickness variations at MAR 23°N by altering source compositions, we performed forward modeling of partial melting of the asthenosphere with variable prior melt depletion relative to fertile DMM [defined as source depletion (F_AD_), Note S6]. Two scenarios were modeled to evaluate the potential variations in the final melting depth (*H*_f_); Model 1 assumes a constant *H*_f_ (= 19.5 km), while Model 2 allows *H*_f_ to vary from 24 km to 15 km. Both modeled scenarios demonstrate that the low magma supply but relatively refractory peridotite compositions for the Kane OCC can be reproduced by upwelling of depleted asthenosphere with F_AD_ of 7%–9% (Fig. 6a). In contrast, the thick crusts during the symmetric spreading stage are consistent with upwelling of more fertile asthenosphere with F_AD_ of 2%–3% (Fig. 6b).

### Implications for crustal production and spreading modes

Spreading modes at MOR reflect the interplay among spreading rate, axial lithosphere thickness, and magma supply, which together regulate the balance between magmatic accommodation and faulting during plate separation [3,55–57]. Magma flux exerts a primary control on spreading modes [3,56]: low magma flux yields insufficient dike intrusion to accommodate plate separation, leading to the formation of large-offset detachment faults. When magma flux is high, the full magmatic accommodation suppresses axial faulting [3,56,58]. Spreading rate governs magma flux and thereby dictates spreading modes: fast-spreading ridges sustain sufficient magma flux to form symmetric spreading and axial high [3]; slow-spreading ridges display variable magma flux, resulting in secular and spatial variations in spreading modes [59,60]. Given that axial lithosphere thickness is primarily determined by magma supply, recent models build relationships among spreading modes, crustal thickness, and spreading rate (Fig. S10), successfully accounting for the diversity of global MOR with minor exceptions [56,57]. These models suggest that asymmetric spreading occurs when crustal thickness is <3.5 km at a full spreading rate of 25 mm yr^−1^ (Fig. S10). Our forward modeling shows that upwelling of the asthenosphere with F_AD_ of >5% yields sufficiently low magma flux to promote detachment faulting (Fig. S11).

In support of this framework, ancient melt depletion has been reported for the Kane OCC (Fig. 2) as well as several other OCCs along the MAR, e.g. 14°–17°N [14,23], Atlantis Massif [27]. These OCCs maintain low magma flux and refractory peridotite compositions [14,23], inconsistent with melting of a fertile DMM source (Fig. S12). Our results and previous studies [23] reveal that peridotites from these OCCs record Re-depletion ages considerably older than the overlying oceanic crust (Fig. 2b), implying a link between asymmetric spreading and upwelling of ancient refractory mantle. If this connection holds, the degree of ancient melt depletion in the asthenosphere may be more extensive than previously anticipated [19,20], providing the ubiquitous occurrence of asymmetric spreading along slow-spreading ridges [4].

Moreover, our study at MAR 23°N documents that the magma-starved asymmetric spreading phase can be terminated by the arrival of more fertile mantle, triggering a transition to symmetric spreading (Fig. 6). Thus, mantle heterogeneity can play a crucial role in modulating spreading modes along slow-spreading ridges, underscoring the complex interplay between deep mantle compositions and shallow lithospheric dynamics [7,10,11].

## MATERIALS AND METHODS

### Kane OCC peridotites, cumulates, and basalts

In this study, 30 residual mantle peridotites, 7 gabbros, and 11 basalts were analyzed for geochemical and isotopic measurements. These samples were collected from Knorr Cruise 180, Leg 2 (November to December, 2004) at the Kane OCC (23°30′N, 45°20′W), which is ∼30 km west of the MAR axis and just south of the Kane TF [36]. The peridotites were collected mostly from the Cain and Adam domes. The basalts were collected from eight different sites on the footwall of the Kane OCC. The gabbros were all sampled at site JAS 117 from the Adam Dome. Regional geology and detailed sample information are provided in Note S1.

### Reconstruction of magma flux at MAR 23°N over the past 3.3 million years

The size and thickness of gabbro bodies in the Kane OCC are constrained by tomographic structural models (Note S7) [24], which are utilized to calculate the average thickness of the lower oceanic crust (*H*_LC_), yielding values of 0.1–0.3 km and 0.3–0.6 km for WKO and EKO, respectively. The average thickness of the conjugate upper crust (*H*_UC_) is calculated based on the seismic image of ref. [5], i.e. the upper crust [P-wave velocity (Vp) <6.4 km/s, ref. [61]] with magnetic anomalies 2 to 2A, yielding values of 2.1 ± 0.4 km. For the asymmetric segment, the magma flux (φ) can be estimated following the model of ref. [30]:


(1) \begin{eqnarray*}
\varphi = {H}_{{\rm conj}} \times {U}_{{\rm conj}} + {H}_{LC} \times {U}_{\rm f},
\end{eqnarray*}

where *H*_conj_ and *U*_conj_ are the thickness and spreading rate of the conjugate crust [*U*_conj_ = 11.3 mm yr^−1^, ref. [62]], *U_f_* is the spreading rate of the footwall [*U_f_* = 14.1 mm yr^−1^, ref. [62]]. As the model assumes gabbro accretion equally between the OCC sides [30], *H*_conj_ is expressed as follows:


(2) \begin{eqnarray*}
{H}_{{\rm conj}} = {H}_{UC} + {H}_{LC}.
\end{eqnarray*}

The initial igneous crustal thickness (*H*_c_) can be computed from magma flux (*φ*):


(3) \begin{eqnarray*}
H{\rm c} = \frac{\varphi }{{{U}_{{\rm conj}} + {U}_f}}.
\end{eqnarray*}

These calculations yield similarly low magma flux and igneous crustal thickness for the WKO (φ = 26–29 km^2^ Myr^−1^, *H*_c_ = 1.0–1.2 km) and EKO (φ = 31–38 km^2^ Myr^−1^, *H*_c_ = 1.2–1.5 km, Table S6).

The *H*_c_ value of the RTI Massif cannot be well constrained due to a lack of high-resolution seismic data and only a qualitative estimate can be obtained based on numerical simulation [30] and seismic results [5]. Previous numerical simulation suggests a positive correlation between the proportion of gabbros exposed on the footwall of a detachment fault and magma supply [30]. Compared to the Kane OCC (Fig. 1b), the RTI Massif has a higher proportion of gabbros exhumed on its footwall [63], implying a higher magma flux during its formation [30]. Therefore, the RTI Massif is inferred to have *H*_c_ of >1.5 km. Moreover, previous seismic investigations show the seismic Moho has a depth of 2.5 km for the volcanic seafloor on the top of the RTI Massif [5]. Nonetheless, the possibility of a serpentinized layer, seismically indistinguishable from gabbros [64], suggests that the original igneous crust should not be thicker than 2.5 km. Thus, the RTI Massif is suggested to have *H*_c_ of 1.5–2.5 km, yielding a low magma flux (φ) of 38–63 km^2^ Myr^−1^.

Seismic results suggest a thick oceanic crust below the axial valley (∼5.5 km, Fig. S1a). However, the velocity structure 4.0–5.5 km below the axial valley was poorly constrained due to low seismic-ray coverage, i.e. the normalized derivative weight sum (DWS) <0.3 (Fig. S1b). Applying the Vp structure for the regions with DWS >0.3, the oceanic crustal thickness of the axial valley is >4.0 km (Fig. S1a, b). For the symmetric segment, the magma flux (φ) is described as


(4) \begin{eqnarray*}
{\boldsymbol{\varphi }} = {{\boldsymbol{H}}}_{\boldsymbol{c}} \times {{\boldsymbol{U}}}_0,
\end{eqnarray*}

where *U*_0_ is the full spreading rate [30]. The thick crust below the axial valley (4.0–5.5 km, Fig. 1c) suggests a high magma flux of 100–138 km^2^ Myr^−1^.

### Melt-PX modeling

Melt-PX modeling was conducted to examine whether melting a fertile DMM-like source can account for the low original crustal thickness (*H*_c_) and high melting degrees based on peridotites (F_per_) for the Kane OCC and RTI Massif. The Melt-PX simulation links F_per_, pyroxenite proportion, and final melting pressure [12,45,65]. Following the approach described in ref. [12], a fertile lherzolite composition (15% modal Cpx; ref. [20]) was blended with variable proportions (0 to 0.4) of pyroxenite (M7-16), and decompression melting was simulated across a final melting pressure of 1.3 to 0.5 GPa and *T*_p_ of 1350^o^C and 1300^o^C. The model predicts crustal thickness as a function of F_per_, pyroxenite fraction, and final melting pressure [12,45] (Fig. 4). At fixed F_per_, increasing either the final melting pressure or the pyroxenite fraction elevates predicted crustal thickness, while absent pyroxenite yields a minimum oceanic crustal thickness scenario (Fig. 4a, b). Our simulations reveal that the observed *H*_c_ for the Kane OCC and RTI Massif both fall below the theoretical minimum for melting of a fertile DMM-like source, irrespective of the assigned *T*_p_ or final melting pressure (Fig. 4a, b).

To reconcile this mismatch, we now consider a mantle source with variable degrees of prior melt depletion. We modeled Melt-PX simulation by assuming no pyroxenite in the source for simplicity, fixing *T*_p_ of 1350^o^C, and changing the initial peridotite compositions from the fertile mantle (FM) [20] to a refractory mantle after up to 17% melting. Other input parameters are similar to the modeling of fertile peridotite plus pyroxenite. The modeling results show that ∼8% prior melt depletion relative to FM source is required to reproduce the observed *H*_c_–F_per_ pairs for the Kane OCC and RTI Massif (Fig. 4c). Such depletion likely corresponds to a mantle source less depleted than the present-day DMM [19], given Earth’s progressive asthenospheric melt extraction over geological time [44,47,48]. We interpret this ∼8% depletion as a conservative minimum for the Kane OCC mantle source.

## Supplementary Material

nwaf385_Supplemental_Files

## DATA AND MATERIALS AVAILABILITY

All data needed to evaluate the conclusions, and supplementary codes are available in the Science Data Bank (https://doi.org/10.57760/sciencedb.24623).
