# Supplementary material for: Spreading modes at slow-spreading ridges shifted by mantle heterogeneity of the asthenosphere
Source: Natl Sci Rev. 2025 Sep 11;12(11):nwaf385. doi: 10.1093/nsr/nwaf385 (PMC12576954; doi:10.1093/nsr/nwaf385)
Supplement: nwaf385_Supplemental_Files [file nwaf385_supplemental_files.zip › Supplementary Codes_2509/Supplementary Codes_2502/Supplementary Codes_2505/Fig. 6/Model/Kane_code_readme.pdf]

Modelling crustal production at Kane fracture zone

Author: Boda Liu

Date: 2024/07/29

Email: bodaliu@mail.iggcas.ac.cn

1. Get ready
    1) Make sure you have a C++ compiler. I use g++ in Windows Subsystem for Linux. There are many tutorials, e.g. https://code.visualstudio.com/docs/cpp/config-wsl
    2) We need to install boost library (https://www.boost.org/). Please follow the steps in the official documentation. The version I use is 1.71.0.0ubuntu2.
    3) We need to enable openmp parallel computation. You can add flag to the compiler. In visual studio, I add one line "-fopenmp" in task.json

```
.vscode > {} tasks.json > [ ] tasks > {} 0 > [ ] args
{
    "tasks": [
        {
            "type": "cppbuild",
            "label": "C/C++: g++ build active file",
            "command": "/usr/bin/g++",
            "args": [
                "-fdiagnostics-color=always",
                "-g",
                "${file}",
                "-o",
                "${fileDirname}/${fileBasenameNoExtension}",
                "-fopenmp",
                "-I/usr/local/include",
                "-L/usr/local/lib",
                "-lboost_system",
                "-pthread",
                "-I", "/home/boda/projects/Kane_mantle_array"
            ],
```

2. Input data, set up the model
    1) To change input data, modify lines starting from #27 inside namespace "data_namespace"

```
27 namespace data_namespace {
28     const int data_size = 6;
29     const double x_data[] = {72.3294, 61.2018, 50.0742, 22.2552, 16.6914, 0};
30     const double data[] = {1.10, 1.13, 1.36, 2.00, 4.75, 4.75};
31     const double sigma[] = {0.07, 0.10, 0.14, 0.50, 0.75, 0.75};
```

where x_data is the distance away from the axis, data is the crustal thickness in km.

2) To change the final melting depth, change lines 22-23 in project_constants.h

```
22  const double h_min = 19.5; // corrsponds to Fmax = 13.5
23  const double delta_F = 0.0; // default 0, or 0.03
```

3. Specify parameters in Monte Carlo sampling

1) To change the number of Gibbs steps, modify line 14 in project.h

```
14  const int MCMC_length_this_global = 10000;
```

2) To change the number of parallel threads, modify line 13 in MCMC.h

```
13  const int use_thread_number = 60;
```

3) You can also change MCMC algorithms by modifying line 16 in MCMC.h

```
15  enum MCMC_type {Gibbs = 0, MH = 1 };
16  const MCMC_type MCMC_type_global = MH;
```

4. Set up output file

Specify the output folder in line 15 of project_constants.h. Here, I save the output in disk D of the Windows system. Please keep the default filename, “mcmc_x_Kane_mantle”.

```
15  string output_file_format{ "/mnt/d/local_project/Kane_mantle_array/output/mcmc_x_Kane_mantle" };
```

5. Run

Compile the main cpp file (hellowworld.cpp) and run. Outputs are model parameters and cost in each line. The execution time for my 64-core workstation is a couple of hours. Of cause the time depends on the computation power, Gibbs steps, and the number of models to search in each step.

```
0 9.76e-02 4.70e-02 4.07e-01 1.16e-01 1.39e-01 4.09e-01 1.17e-01 1.64e-01 2.15e-01 2.16e-01 1.90e-01 2.14e-01 cost = 2.34
1 9.83e-02 1.30e-01 4.65e-01 2.94e-01 2.85e-01 2.40e-01 9.02e-02 2.13e-01 2.07e-01 1.88e-01 1.70e-01 2.44e-01 cost = 2.22
2 9.45e-02 6.07e-04 6.17e-01 2.33e-01 4.03e-01 2.45e-01 4.58e-02 2.44e-01 1.74e-01 1.67e-01 1.61e-01 2.35e-01 cost = 2.13
3 9.27e-02 1.22e-01 5.68e-01 4.04e-01 3.22e-01 3.53e-01 5.85e-02 2.19e-01 1.64e-01 2.15e-01 1.92e-01 1.99e-01 cost = 1.97
4 9.35e-02 8.36e-03 5.14e-01 4.64e-01 1.12e-01 4.50e-01 2.69e-02 2.32e-01 1.24e-01 2.41e-01 1.94e-01 1.90e-01 cost = 1.79
5 9.53e-02 8.15e-02 5.69e-01 6.11e-01 2.91e-01 3.25e-01 6.69e-02 2.43e-01 1.64e-01 2.84e-01 1.65e-01 2.21e-01 cost = 1.40
6 9.25e-02 4.00e-03 6.29e-01 8.04e-01 1.58e-01 2.42e-01 3.77e-02 2.73e-01 1.21e-01 2.44e-01 1.80e-01 1.95e-01 cost = 0.98
7 9.28e-02 1.80e-01 3.82e-01 6.40e-01 3.49e-01 1.62e-01 2.43e-02 2.58e-01 1.65e-01 2.31e-01 2.11e-01 1.78e-01 cost = 1.71
8 9.64e-02 2.68e-01 5.93e-01 8.24e-01 1.75e-01 2.79e-01 3.32e-02 2.13e-01 1.24e-01 1.91e-01 2.47e-01 2.10e-01 cost = 1.21
9 1.02e-01 2.75e-01 3.71e-01 9.44e-01 2.47e-01 3.38e-01 5.63e-03 1.91e-01 1.33e-01 1.81e-01 2.05e-01 2.17e-01 cost = 4.09
```

…

```
Write to the file: /mnt/d/local_project/Kane_mantle_array/output/NEW_mcmc_MH_Kane_mantle_tg_hmin_19.5

Calculate properties...
Done!

Write to the file: /mnt/d/local_project/Kane_mantle_array/output/NEW_mcmc_property_Kane_mantle_tg_hmin_19.5
```
