# Supplementary material for: Spreading modes at slow-spreading ridges shifted by mantle heterogeneity of the asthenosphere
Source: Natl Sci Rev. 2025 Sep 11;12(11):nwaf385. doi: 10.1093/nsr/nwaf385 (PMC12576954; doi:10.1093/nsr/nwaf385)
Supplement: nwaf385_Supplemental_Files [file nwaf385_supplemental_files.zip › Supplementary Materials.pdf]

Supporting Information for

Spreading mode at slow-spreading ridges shifted by mantle heterogeneity of the asthenosphere

Wei-Qi Zhang et al.

*Corresponding author. Email: chzliu@mail.iggcas.ac.cn

This PDF file includes:

Supplementary Note 1 – 9

Figs. S1 – S13

Legend for Table S1 to S8

Legend for Supplementary Code

Legend for Supplementary References

Other Supplementary Materials for this manuscript include the following:

Tables S1 to S8

Supplementary Code

Supplementary Note 1 – Regional geology and sample information

The ridge segment at MAR 23°N south of the Kane transform has been extensively investigated [1-6]. The axial valley of the northern segment at MAR 23°N is characterized by active volcanism and symmetrical spreading (Fig. 1b); it has a thick and layered oceanic crust (up to 5.5 km) and melt-present layers (>2 km deep) with an estimated 4% melt [5, 7]. The footwall of a detachment fault (hereafter referred to as the RTI Massif) is exposed at the high-relief ridge-transform intersection 5-30 km off-axis at 23°N [8]. The width of the footwall decreases from north to south along the RTI Massif, ranging from 8-22 km off-axis at 23º36′N to 8-13 km off-axis at 23º30′N [9]. Dive investigations have revealed outcrops of gabbros and minor peridotites along the footwall surface of the RTI Massif [1, 6, 8], 13-24 km off-axis (Fig. 1b). The seafloor magnetic anomaly [2] indicates that the RTI Massif was active during 2-0.4 Ma at ~23º36′N and during 1.5-0.4 Ma at ~23º28′N. Volcanic seafloor is exposed at the top of the RTI Massif (Fig. 1b), belonging to a magmatic crust with a seismic thickness of ~2.5 km (Fig. S1a, b) [1, 5, 6, 8]. A conspicuous eastward dipping of the eastern slope surface of the RTI Massif marks the contact between the rift valley volcanic terrain and the RTI Massif (Fig. 1b, Fig. S1a) and the renewal of robust magmatism that cut off the RTI detachment, as supported by the abrupt and sub-vertical gradient of the seismic Moho boundary near this contact [5, 8].

The Kane OCC is located ~30-55 km off-axis (Fig. 1) and was generated by detachment faulting between 3.3 and 2.1 Ma [4]. An eastward ridge jump at ~2 Ma is suggested by the absence of the magnetic anomaly 1r.3r on the eastern ridge flank but its presence on the western flank between the Kane OCC and the RTI Massif [2]. After the ridge jump, the RTI detachment was initiated shortly after the termination of the Kane OCC. Asymmetric spreading between 3.3 and 0.4 Ma resulted in the sequential development of two detachment systems [2, 8], after which the MAR at 23°N was transitioned to magmatically-robust symmetric spreading at 0.4 Ma (Fig. 1, Fig. S1a, b).

The Kane OCC has been extensively mapped by seafloor sampling and seismic investigations, revealing a highly heterogeneous distribution of gabbro bodies and mantle on the footwall (Fig. S1b). Seismic results indicate that limited gabbros occur in the upper 2 km in the Abel Dome and Mt.Ararat, which together with Adam Dome, form the Western part of Kane OCC (WKO) [4]. Half of the exposed basement at Adam Dome is composed of gabbro, indicating the presence of a ~40 km^2 gabbro body with an estimated thickness of 0.5-1.0 km [4,

10]. The Eastern part of the Kane OCC (EKO) consists of Eve Dome, Babel Dome, and Cain Dome; seafloor sampling and seismic imaging reveal a >81 km^2 gabbroic crust ~1 km thick [4].

The mantle peridotites from the Kane OCC selected for this study consist mainly of harzburgites with <5 vol% modal Cpx, with only two lherzolites (6-7 vol% Cpx). Magmatic veins are absent from the hand specimens. They are characterized by protogranular to porphyroclastic textures (Fig. S2a). They are pervasively serpentinized, with olivine completely altered to serpentine and pyroxenes partially replaced by serpentine or tremolite. The high degrees of alteration hinder estimating of their primary mineral modes by point counting. Their CIPW (acronym from the surnames of the authors: Cross, Iddings, Pirrson and Washington) mineral modal contents were calculated using the approach of ref. [11], before correcting for the effects of carbonation using the method of ref. [12]. Whole-rock compositions were recalculated to 100 wt% anhydrous for the CIPW calculation.

Supplementary Note 2 – Analytical Methods

Whole-rock major and trace elements. Whole-rock major and trace elements were measured at the Institute of Geology and Geophysics, Chinese Academy of Sciences (IGGCAS). LOI was measured by the weight difference before and after heating to 1,000ºC. About 0.6 g of 200-mesh sample powder was weighed and mixed with 6 g of Li_2BO_7, before the glass beads were made. The glass beads were measured on an AXIOS mineral spectrometer, with analytical uncertainties of 1-3%. According to X-ray fluorescence measurements of USGS standards GSR-1, GSR-3 and an ultramafic standard JP-1 (Table S1), the external uncertainties are 0.1-0.2 wt% for SiO_2 and <0.02 wt% for Fe_2O_3, MgO, Na_2O, K_2O, TiO_2, MnO, CaO, and P_2O_5. For trace elements, forty milligrams of powders were weighed and digested in a 1:2 mixture of 50% HNO_3 and HF for >120 h at 240ºC in an oven. Rock standards (JP-1, DTS-2B, and BHVO-2) and blanks were prepared along with the samples. After drying and subsequent 50% HNO_3 desiccation steps to remove fluorides, sample residues were dissolved in 20% HNO_3 at 150ºC for >6 hours. These solutions were then diluted 1250 times in ultrapure water. The solutions were measured utilizing a Thermo Fisher iCAP RQ quadrupole inductively coupled plasma mass spectrometer (ICP-MS). The reference materials showed an overall reproducibility of better than 5% (RSD) and their elemental contents were consistent with the recommended values (Table S1).

Mineral major and trace elements. Mineral major elements were analyzed on a JEOL JXA-8100 Electron Probe at the IGGCAS, using an acceleration voltage of 15 keV, a beam current of 10 nA and beam size of 3 to 5 μm. Mineral trace element abundances were determined by laser ablation-ICP-MS employing an Element XR HR-ICP-MS instrument (Thermo Fisher Scientific, USA) coupled to a 193 nm ArF excimer laser system at the IGGCAS, using a peak-hopping mode with a laser diameter of 32 to 60 μm and repetition rate of 5 Hz. The laser energy density was 4.0 J/cm^2. ARM-3 reference glass was used for external calibration and BIR-1G glass was used for quality control monitoring. Silicon (^{29}Si) was used as an internal standard. For most trace elements (>0.005 $\mu g\ g^{-1}$) the accuracy is better than ±10 % with an analytical precision (1 RSD) of ±10%.

Highly siderophile elements (HSE) and Os isotopes. Whole-rock HSE and Os isotopes were analyzed at the IGGCAS utilizing the isotope dilution method. Two grams of sample powder and appropriate amounts of a ^{187}Re-^{190}Os mixed spike and a ^{191}Ir-^{99}Ru-^{194}Pt-^{105}Pd mixed spike were weighted into a Carius Tube, together with 9 ml of 1:2 mixed HCl: HNO_3 acids at 220°C for three days, Os was extracted from the solution with CCl_4 and then back-extracted into HBr. Os was then purified through micro-distillation. Re, Ir, Ru, Pt and Pd were separated and purified by anion exchange chromatography using 200 meshed AG 1×8 resin into Re-Ru, Ir-Pt, and Pd fractions. The Re-Ru fraction and the Ir-Pt and Pd fractions were re-purified using a small anion exchange column and an Eichrom LN spec resin, respectively. Finally, each Re-Ru, Ir-Pt, and Pd fraction was dried and re-dissolved in 0.8 N HNO_3 before measurements by a Thermo Fisher iCAP RQ ICP-MS. The in-run precisions for $^{185}Re/^{187}Re$, $^{191}Ir/^{193}Ir$, $^{99}Ru/^{101}Ru$, $^{194}Pt/^{196}Pt$ and $^{105}Pd/^{106}Pd$ were <0.3% (2RSD). The Re, Ir, Ru, Pt and Pd standard solutions were utilized for mass fractionation correction. The 20 ppb Hf, 20 ppb Zr, and 50 ppb Cd standard solutions were utilized for interference corrections, i.e., $^{90}Zr^{16}O^+$ on $^{106}Pd^+$, $^{177}Hf^{16}O^+$ on $^{193}Ir^+$, $^{178}Hf^{16}O^+$ on $^{194}Pt^+$, $^{180}Hf^{16}O^+$ on $^{196}Pt^+$, and $^{106}Cd^+$ on $^{106}Pd^+$. Os contents and isotopic ratios were determined on a Thermo Fisher Triton Plus instrument in static mode using Faraday cups. $Ba(OH)_2$ solution was used as an ion emitter. The measured Os isotopes were corrected for mass fractionation using the $^{192}Os/^{188}Os$ ratio of 3.0827. The Nier oxygen isotope composition ($^{17}O/^{16}O$ = 0.0003708 and $^{18}O/^{16}O$ = 0.002045) was used for the oxide correction. The in-run precisions for Os isotope measurements were better than 0.2%. The standard of UMD was utilized as an external standard, yielding a $^{187}Os/^{188}Os$ ratio of 0.11378 ± 2 (1σ; n = 7). The

standard UB-N yields results of 3.1±0.5 ppb for Ir, 6.3±0.6 ppb for Ru, 7.1±0.3 ppb for Pt, 6.1±0.2 ppb for Pd, 0.22±0.02 ppb for Re (1σ, n=9), 3.3±0.4 ppb for Os, and 0.1276±5 for $^{187}Os/^{188}Os$ (1σ, n=8), consistent with reported values (Table S3). The total procedural blanks are 2.4±2.7 pg for Os, 4.0±2.1 pg for Ir, 15±7 pg for Ru, 34±12 pg for Pt, 23±6 pg for Pd, 4.0±0.8 pg for Re.

Whole-rock and clinopyroxene Sr-Nd isotopes. Sr-Nd isotopes were measured at Wuhan Sample Solution Analytical Technology Co., Ltd., Wuhan, China. For cumulates, the separated Cpx was leached three times before chemical digestion. After centrifugation, the supernatant solution was loaded into an ion-exchange column packed with AG50W resin. After complete draining of the sample solution, the columns were rinsed with 2.5 M HCl to remove undesirable matrix elements. Finally, the Sr fraction was eluted with 2.5 M HCl and gently evaporated to dryness prior to mass-spectrometric measurement. The residue was rinsed with 10 mL of 4.0 M HCl and the REE fraction was eluted with 10 mL of 4.0 M HCl. The REE solution was evaporated to incipient dryness and taken up with 0.18 M HCl. The converted REE solution was loaded into an ion-exchange column packed with LN resin. After complete draining of the sample solution, columns were rinsed with 0.18 M HCl to remove undesirable matrix elements. Finally, the Nd fraction was eluted with 0.3 M HCl and gently evaporated to dryness prior to mass-spectrometric measurement. Sr-Nd isotope analyses were performed on a Thermo Fisher Neptune Plus MC-ICP-MS. The large dry interface pump (120 m^3 hr^{-1} pumping speed), and newly designed H skimmer cone and standard sample cone were used to increase the instrumental sensitivity. Alfa Sr and Alfa Nd standard solutions optimized instrument operating parameters. NIST SRM 987 and GSB 04-3258-2015 were used to evaluate the reproducibility and accuracy during the Sr and Nd isotope measurements, respectively. Analyses of the NIST 987 and GSB standard solutions yielded an $^{87}Sr/^{86}Sr$ ratio of 0.710244±1(2SD, n=10) and $^{143}Nd/^{144}Nd$ ratio of 0.512439±4 (2SD, n=10), respectively, consistent with the published values (Table S4). The standard JB-3 and RGM-2 yielded results of 0.703414±5 for $^{87}Sr/^{86}Sr$ and 0.513067±9 for $^{143}Nd/^{144}Nd$ and 0.704143±6 for $^{87}Sr/^{86}Sr$ and 0.512803±10 for $^{143}Nd/^{144}Nd$, respectively, consistent with their published values (Table S4).

Supplementary Note 3 – Effects of alteration on the geochemistry and Os isotopes of peridotites

The Kane OCC peridotites exhibit petrologic features of strong serpentinization (Fig. S2a). The high loss on ignition (LOI) of 3-14 wt% and high U/Th ratios of 0.3-164 in Kane OCC peridotites indicate serpentinization (<400ºC) and/or seafloor weathering (~0ºC). Serpentinization can increase fluid mobile elements (U, Sr, Na, K, Sr, LREE) [11] and decrease Mg, Al_2O_3, but high field strength elements (HFSE, Zr-Hf-Ti-Nb-Ta-Th) and heavy rare earth elements (HREE) are resistant to alteration processes [11]. Re and Pd can be mobile at thin-section scale during serpentinization [13], but whole-rock Re and Pd contents of abyssal peridotites remain largely unchanged by such processes [14, 15]. U/Th ratios, an indicator of seawater addition, do not correlate with Re, Pd, Pt, and Os abundances or $^{187}Os/^{188}Os$ in Kane OCC peridotites (Fig. S2c-f), implying that alteration has little effect on their whole-rock HSE abundances and Os isotopes. Binary mixing modeling between peridotites and seawater also indicates that the peridotite $^{187}Os/^{188}Os$ remain unmodified even at water/rock ratio up to ~700 (Fig. S2e).

Moreover, Day et al. [16] have shown that variously altered peridotites from the same sampling sites of Tonga forearc exhibit similar ranges of whole-rock Al_2O_3 contents, HSE contents, Os/Ir and Ru/Ir ratios, and $^{187}Os/^{188}Os$ ratios. Likewise, measurements of the altered rims and fresh cores of Gakkel Ridge peridotites suggest that their HSE abundances and $^{187}Os/^{188}Os$ ratios are unaffected by low-temperature alterations [15, 17]. These observations and previous works [14-17] suggest that HSE abundances and Os isotopes in Kane OCC peridotites have preserved their original mantle features.

Supplementary Note 4 – Geochemical modeling of partial melting of ancient refractory mantle

Compositions of ancient refractory mantle. Ancient refractory mantle (RM) within the asthenosphere is typically regarded as recycled oceanic lithosphere [18, 19]. This recycled lithosphere originated from ancient melting within a triangular melting region (Fig. S8a), resulting in a bottom-up melt depletion gradient. Following the methodology of ref. [19], the bulk compositions (Nb, La, Zr, Sm, Yb) of RM were reconstructed as a weighted sum of single melting intervals (1% melting) from a DMM-like mantle residual in the triangular melting

region. By applying a maximum ancient melting degree of 24%, our reconstructed RM exhibits bulk heavy rare earth element (HREE) contents (e.g., Yb) similar to residues after 7–8% melting of DMM (Fig. S8a).

To further assess the fertility of RM, we estimated bulk Al_2O_3 contents—a parameter sensitive to source fertility [20]. Previous studies have shown that during low-pressure spinel-facies melting beneath ocean ridges, Al_2O_3 and Yb exhibit similar incompatibility, evidenced by their strong correlation in residual abyssal peridotites [21], i.e., Al_2O_3 (wt.%) =10.788×Yb (μg g^{-1}), R^2=0.84 (Fig. S8b). Using this relationship, we determined that the Yb contents in RM and DMM correspond to Al_2O_3 abundances of 2.2 wt% and 4.3 wt%, respectively. The latter aligns with the recommended DMM value [22], confirming the reliability of this estimation. Accordingly, the RM component contains 0.0015 μg g^{-1} Nb, 0.003 μg g^{-1} La, 0.975 μg g^{-1} Zr, 0.060 μg g^{-1} Sm, 0.205 μg g^{-1} Yb, and 2.2 wt.% Al_2O_3, indicating a fertility similar to 7 to 8% melting residues of DMM. This fertility is consistent with numerical simulations of mantle melting and crust production, which require 7–9% ancient melt depletion during the asymmetric spreading stage of the Mid-Atlantic Ridge at 23°N (Fig. 6a).

Melting model. We calculated the trace element compositions of magmas using a dynamic melting model, as described in refs. [19, 22]. We set a residual porosity of 0.02% and extracted 0.2% melt at each step, with a fixed melting rate of 0.2% per kilometer. Initial compositions of DMM, partition coefficients, and melting equations were sourced from ref. [22]. For DMM melting, we assumed 3% melting occurs in the garnet stability field, with greater degrees in the spinel stability field. For RM melting, all melting occurred in the spinel stability field.

Our results showed that aggregated melts from low-degree RM melting were too depleted in highly incompatible elements and had excessively high Zr/Nb ratios compared to the primary melts of Kane OCC MORBs. Therefore, we considered partial melting of sources mixing RM with minor amounts of DMM (4% and 10%). Model input parameters and results are given in Table S7.

Supplementary Note 5 – Reconstruction of primary melts of Kane OCC and axial valley MORB

As the geochemical modeling of mantle melting only predicted the compositions of primary melts, it is necessary to reconstruct the trace element compositions of the Kane OCC and axial

valley MORB back to their corresponding primary melt compositions. This reconstruction was performed following the approach similar to that detailed in ref. [23]. During MORB fractional crystallization, MgO concentration in residual melts is generally linearly correlated with the extent of fractional crystallization and a MgO content of 9.5 wt.% corresponds to a primary melt composition with close to zero degree of fractional crystallization [23]. Similar to the method described in ref. [23], the primary melt trace element (Nb, La, Zr, Sm, Yb) compositions at MgO of 9.5 wt.% were constrained based on the linear correlations between MgO contents and the log [M], where [M] is the content of trace element M in MORB (Fig. S9). The primary melt compositions of the Kane OCC and axial valley MORB are given in Table S7.

Supplementary Note 6 – Inferring source depletion using modeling of mantle melting and magma production

We performed numerical simulations of decompression melting of a heterogeneous asthenosphere consisting of variable refractory mantle (RM) and fusible components. Due to limited constraints on the forms and compositions of these components, we utilized the mean mantle depletion (F_{AD}), the averaged prior melting extents relative to a fertile mantle (FM) source with major element compositions analogous to DMM [24], to describe mantle compositional variations below MAR 23°N. In this study, we consider the passive flow model and melting was considered to occur within a triangular melting region, as depicted in figure 2d of ref. [25]. First, maximum melting degrees recorded by abyssal peridotites are 16–18% for most ocean ridges [26], suggesting that mantle melting terminates below the base of the crust, i.e., near a final melting depth [25, 27]. Second, magnetotelluric resistivity images of ocean ridges consistently reveal a triangular melting region with variable final melting depths ranging from 35 km to 10 km [28-30], likely reflecting variations in axial lithosphere thickness. Based on these observations, we modeled sub-ridge melting within a triangular region, with the maximum melting degree modulated by the final melting depths.

The solidus of FM is a function of the volatile content (H_2O) and the potential temperature (T_p) [31]. For dry FM and a normal T_p of 1350ºC, decompressional melting starts at 60 km (i.e., a dry solidus) [32]. With 100 μg g^{-1} H_2O in FM, melting can commence at depth up to 100 km (the "wet solidus") [33]; however, the degree of melting between the wet solidus and dry solidus is limited (<2%) [33]. Therefore, the initial melting depth of FM is assumed to be 60 km, i.e., the

dry FM solidus. Magnetotelluric resistivity image of the fast-spreading East Pacific Rise reveals a width of 120 km at the depth of dry FM solidus (60 km) [28], reflecting a geometry of melting region formed by passive mantle flow. Similarly, imaging of the ultraslow-spreading Mohns-Konipovich Ridge reveals melting zone width of 90 to 120 km at dry FM solidus [29, 30]. Geodynamic models suggest that such small variations in melting zone widths have negligible impact on modeled crustal thickness [34]. Furthermore, since active mantle flow contributes less than 20% at ridges with full spreading rates of 25 mm yr^{-1} [34, 35], we adopted a similar melting region width of 120 km at the dry FM solidus for our model [28].

The spatial and temporal variations in final melting depths, as indicated by resistivity images [28-30], are crucial in modulating crustal production. Hence, the final melting depth is treated as either a constant value (19.5 km, Model 1) or varying values (from 24 to 15 km, Model 2), which result in different maximum melting degree (F_{FM}^{MAX}). These values for the final melting depths are selected based on the melting degrees recorded by the abyssal peridotites at MAR 23°N (Fig. S7); i.e., based on peridotite data, F_{FM}^{MAX} should vary between 12 and 15% during asymmetric spreading between 3.3 and 0.4 Ma. For simplicity, the melting rates are assumed to be constant with the decreasing pressure. For the asthenosphere having more refractory compositions than FM (i.e., F_{AD}>0%), the upwelling mantle starts to melt at depth of <60 km because its solidus depth depends on the F_{AD}. The variation of the F_{AD} for the mantle source of oceanic crusts accreted during different episodes is treated as a stochastic and monotonically decreasing sequence, i.e., the location and magnitude of the variations in F_{AD} are unknown. A Monte Carlo procedure is used to find the F_{AD} for the asthenosphere upwelled at different times that best reproduces the observed crustal thickness.

We consider the variation of mantle composition with depth at 3.3 Ma. For simplicity and to reduce constraints, the composition structure of the upwelling asthenosphere is assumed to be one dimensional. To calculate the crustal thickness, we apply the integral of melt productivity in the melting region [25],

$$h = \frac{1}{U_0} \int_{H_{lid}}^{H} dz \int_{-z}^{z} dx \frac{dF}{dz} w \tag{5}$$

where U_0 is the full spreading rate; H is the height of the melting region, 60 km; H_f is the final melting depth; w is the upwelling rate, equal to the half spreading rate in a passive mantle flow

model [25], $U_0/2$; the melt productivity dF/dz depends on the depth, the averaged mantle depletion (F_{AD}),

$$\frac{dF}{dz}(z) = \begin{matrix} \frac{F_{FM}^{MAX}}{H - H_f}, z \le z_R \\ 0, z > z_R \end{matrix} \quad (6)$$

where z_R is the solidus of upwelling mantle,

$$z_R = H - (H - H_f)\frac{F_{AD}}{F_{FM}^{MAX}} \quad (7)$$

The forward model as described by these three equations is integrated with the existing algorithm of Markov Chain Monte Carlo method [36] to sample the model parameters (F_{AD}) under the constraints of crustal thickness data (Fig. S11). The best 10000 cases that reproduce the observed crustal thickness variations at MAR 23°N are plotted as violin plots with box-and whisker symbols in Fig. 6. These models demonstrate that the low magma supply but relatively refractory peridotite compositions for the Kane OCC can be reproduced by upwelling of asthenosphere with depleted compositions (F_{AD} =7%–9%, Fig. 6a). In contrast, the thick crusts during the symmetric spreading stage are consistent with upwelling of more fertile asthenosphere with F_{AD} of 2%–3% (Fig. 6b).

Supplementary Note 7 – Reconstruction of the averaged thickness of lower oceanic crust (H_{LC}) for the Kane OCC

The average thickness of the discontinuously exhumed gabbro bodies in the footwall of the Kane OCC was reconstructed utilizing the high-resolution seismic imaging [4]. Xu et al.[4] reconstructed the sizes and distributions of gabbro bodies in the footwall of the Kane OCC. Xu et al.[4] utilized the new approaches of time domain acoustic full wave-form inversion (FWI) and reverse time migration (RTM), which allow the detections of gabbroic intrusions with size of ~0.3 km [37]. Here we describe the methods used to reconstruct the average lower oceanic crustal thickness (H_{LC}) of the Kane OCC including the WKO (between the breakaway zone and the western flank of Cain Dome) and EKO (Babel, Cain and Eve Domes). The results of reconstruction are provided in Table S6.

The WKO. i) Gabbros in Mt. Ararat and Abel Dome: Seafloor sampling results suggest that gabbros are limited underneath Mt. Ararat and Abel Dome [2], implying peridotites are

predominant in this area. Based on the high-resolution seismic imaging, only few gabbro bodies with north-to-south lengths of 0.5-3.0 km and thicknesses of 0.2-0.5 km are present in these areas [4]. As shown in Fig. S13a, along with profiles K1 and K4, no gabbros were detected, suggesting that the west-to-east widths of the gabbros are lower than the resolution (i.e., 0.3 km). Along profile K5, however, several small gabbros with a total length of 5.5 km are detected (Fig. S13a). However, due to the limited seismic profiles measured [4], it is still possible that some small gabbros exist but were not detected along the two W-E-direction profiles (K1 and K4). Therefore, we assumed that the small gabbros underneath Mt. Ararat and Abel Dome have total length width of 5.5 km (equal to the total N-S-direction length of these gabbros) and 0.2 km (the resolution of FWI and RTM approach) for maximum and minimum crustal thickness estimation, respectively. The total length of these gabbros was estimated based on profile K5, yielding a value of 5.5 km (Fig. S13a). The thicknesses of these gabbros are assumed to be 0.2 km and 0.5 km for maximum and minimum crustal thickness estimation, respectively. These treatments result in total gabbro volumes of 0.3 to 15.1 km^3 underneath Mt. Ararat and Abel Dome.

ii) Local magma center at Adam Dome: Limited seismic data are presented here, but seafloor sampling and available seismic data indicate an area of 82 km^2 and a thickness of 0.5-1.0 km for this gabbro body, i.e., profile K5 in ref. [4] suggests a thickness of 0.5 km for the northern part of Adam Dome. Therefore, we consider maximum and minimum thicknesses of 0.5 km and 1.0 km, respectively, for this gabbro intrusion, yielding gabbro volume of 41 to 82 km^3.

The EKO. i) Babel and Cain Dome: Several large gabbros exist underneath the Babel and Cain Dome, showing total width of 6.8 to 9.6 km in the W-E direction (profiles K4 and K1, Fig. S13b), total length of 16.4 km in the N-S direction (profile K7, Fig. S13b), and thickness of ~1 km [4]. Therefore, we consider constant total length of 16.4 km and thickness of 1 km for these gabbros but total width of 6.8 km and 9.6 km for minimum and maximum estimations, respectively. These treatments result in total gabbro volume underneath the Babel and Cain Dome of 112 to 157 km^3. ii) Eve Dome: No seismically detected gabbro bodies are identified in this dome. However, the seafloor sampling suggests ~50% of the basement rocks on the Eve Dome could be gabbros [2]. Here, we consider the gabbros account for ~50% and 0% area of the Eve Dome for the maximum and minimum estimates, respectively. The thickness of the gabbro intrusions below the Eve Dome is assumed to be similar to the Adam Dome, i.e., 0.5 to 1.0 km (ref. [2]).

Supplementary Note 8 – Calculation of the equilibrium melt $(La/Sm)_N$ ratios of the Kane OCC cumulates

Similar to global oceanic gabbros, the Kane OCC cumulates were extensively influenced by melt-rock reactions during the post-cumulus melt evolution. This led to more pronounced fractionation of more-to-less incompatible elements (e.g., La/Sm) in Cpx rims compared to a simple fractional crystallization model [38, 39] but possibly reduced the more-to-less incompatible element ratios in Cpx cores [40]. To minimize the impact of melt-rock reactions, we used the median Cpx $(La/Sm)_N$ ratio in each sample to calculate the equilibrium melt $(La/Sm)_N$ ratios. The Cpx-melt REE partition coefficients were computed based on model of ref. [41]. The Kane OCC cumulate Cpx data and the calculated equilibrium melt compositions are given in Table S8. The results show that the equilibrium melts of the Kane OCC cumulates have a median $(La/Sm)_N$ ratio of 0.56, similar to that of the Kane OCC basalts (0.58) but lower than that of the axial valley basalts (0.65, Fig. S5e).

Supplementary Note 9– Literature data source for figures

Fig. 2: Sources for published data: Leg 153 and Leg 209 [42, 43], Lena [44], and Gakkel [14, 17].

Fig. 3, Fig. 5: Published data for MORB from Mid-Atlantic Ridge at 23°N are downloaded from EarthChem PetDB database and provided in Table S5.

Supplementary Figures

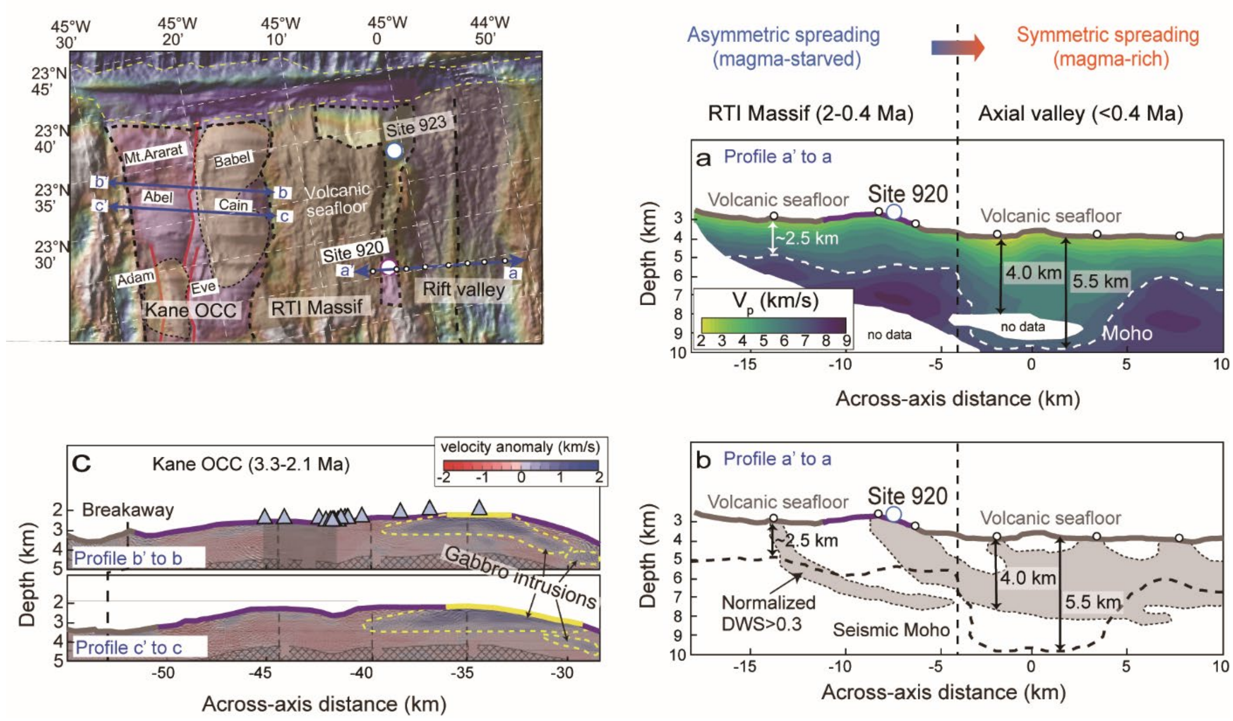

Fig. S1 Velocity structure of MAR 23°N.

a Modified seismic image along profile a'-a across the axial valley and the RTI Massif showing the rapid crustal thickening and transition from asymmetric to symmetric spreading since ~0.4 Ma (ref. [5]). Ocean-bottom seismometer (OBS) locations are marked in open circles in the bathymetric map. **b** Regions with high seismic-ray coverage showed in terms of the normalized derivative weight sum (DWS) along the profile a'-a. **c** Seismic images along two profiles (b'-b and c'-c) illustrating the distribution of gabbro bodies in the Kane OCC [4]. The grey, purple, and yellow lines of seafloor morphology represent the volcanic, ultramafic, and gabbroic seafloors, respectively. Across-axis distances in the two panels are normalized relative to the ridge axis. Locations of profiles a'-a, b'-b, and c'-c are shown in the bathymetric map.

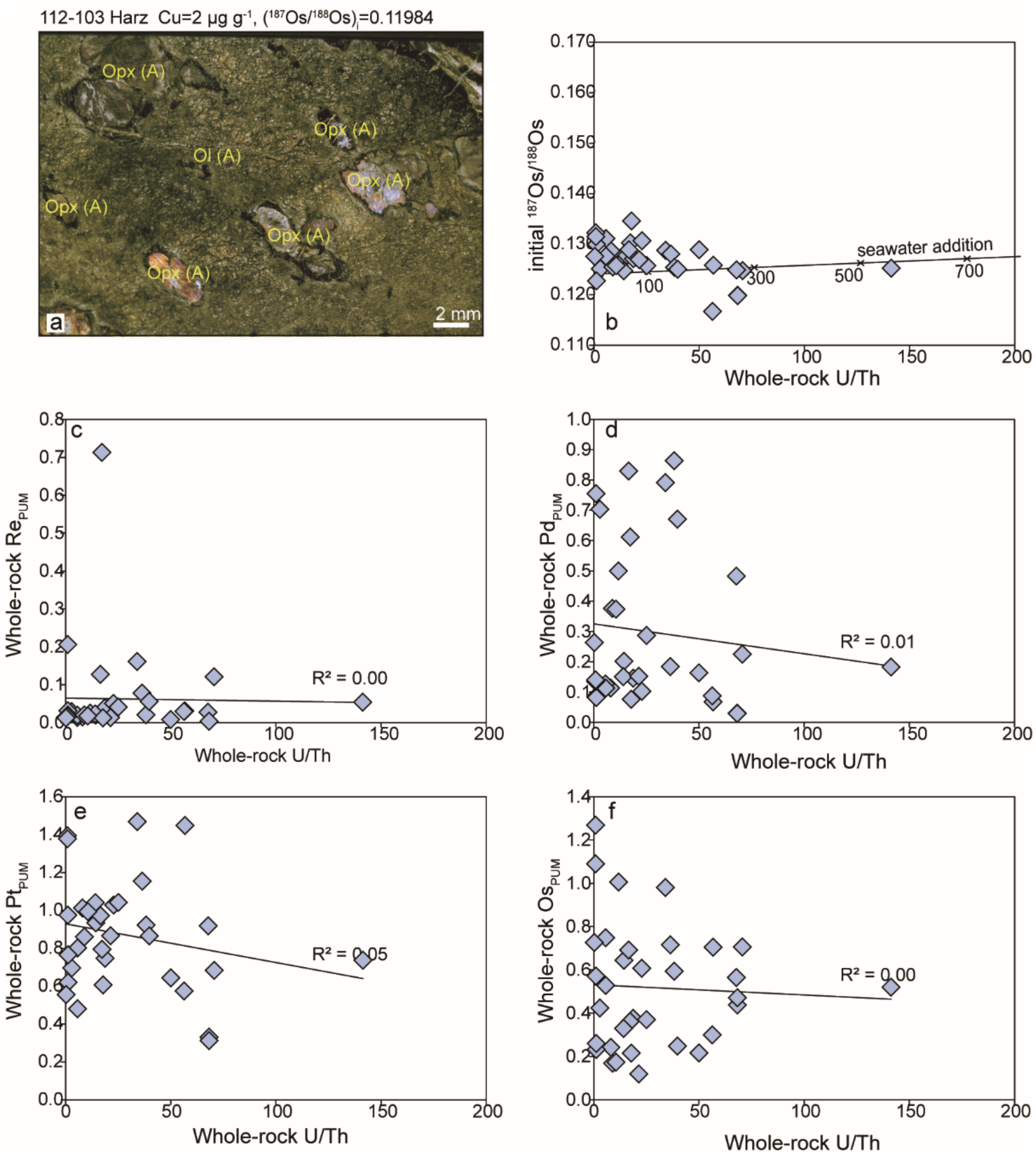

Fig. S2. Effects of alteration on highly siderophile elements and Re-Os isotopes.

a Representative thin section scanning images for the Kane OCC peridotites, with initial $^{187}Os/^{188}Os$ value labeled. Opx=orthopyroxene, Cpx=clinopyroxene, Ol=olivine. (A) indicates that this mineral has been altered. **b-f** Correlations of highly siderophile element abundances [Primitive Upper Mantle (PUM) normalized values, ref. [45]], ratios and Os isotopic compositions with the whole-rock U/Th ratio, a fluid-sensitive indicator, for Kane OCC peridotites. A binary mixing curve between fresh peridotite and seawater is operated for the $^{187}Os/^{188}Os$-U/Th correlation.

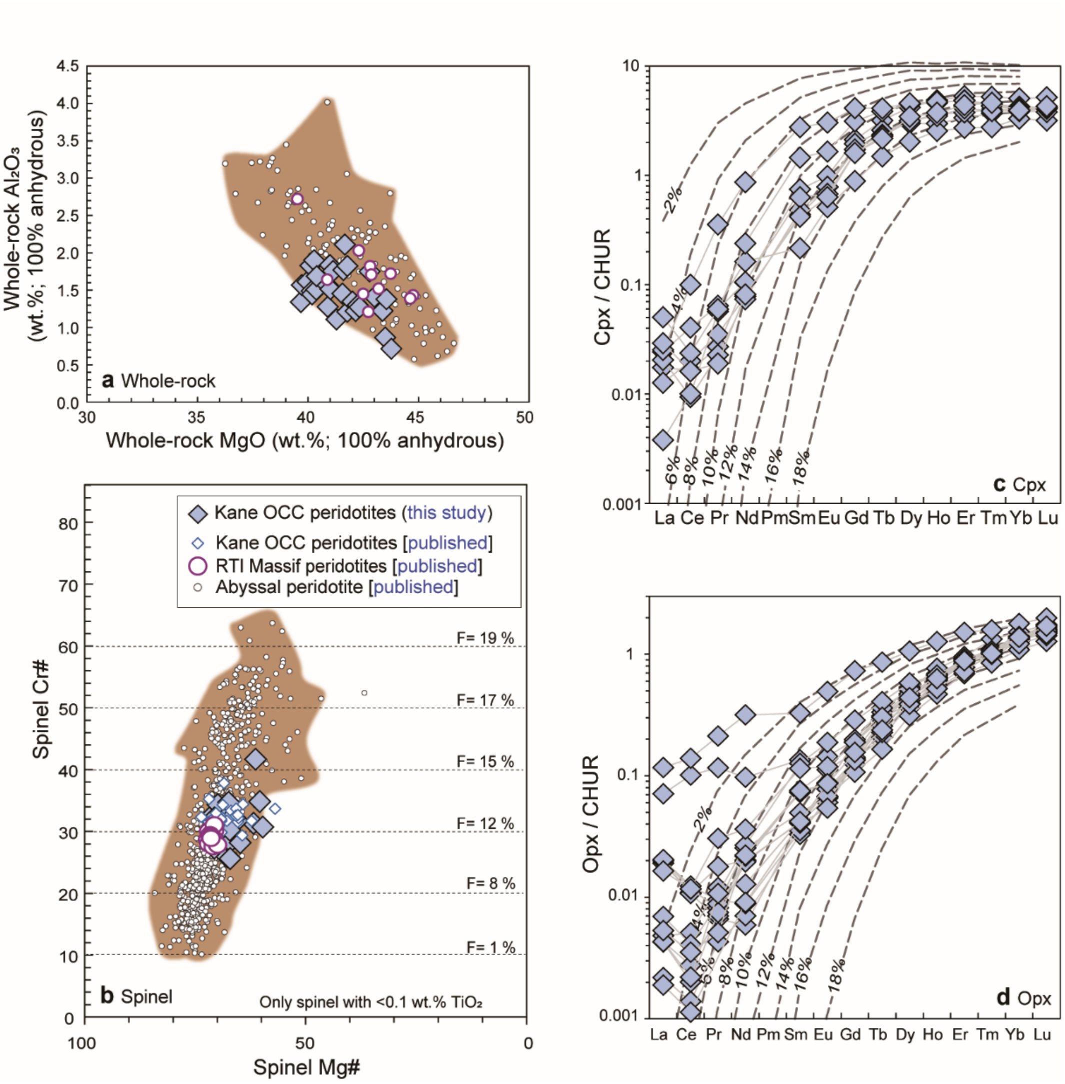

Fig. S3. Whole-rock and mineral geochemistry.

a Whole-rock Al_2O_3 versus MgO (100% anhydrous normalized values). **b** Spinel Cr# versus Mg#. **c-d** CI-chondrite (CHUR) normalized REE patterns for the Kane OCC peridotite clinopyroxene (Cpx), and orthopyroxene (Opx). Data for abyssal peridotites are from ref. [26]. Additional Kane OCC are from ref. [46] and ref. [10]. Melting degrees are calculated based on ref.[47] and refs. [1, 2, 9, 26].

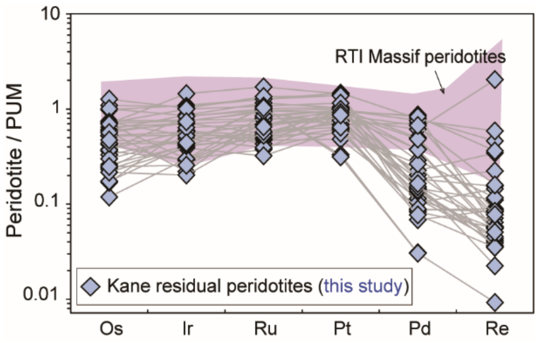

Fig. S4. **Highly siderophile element patterns of the Kane OCC peridotites.**

PUM values are taken from Becker et al. [45]. Sources of the published data are provided in Table S3.

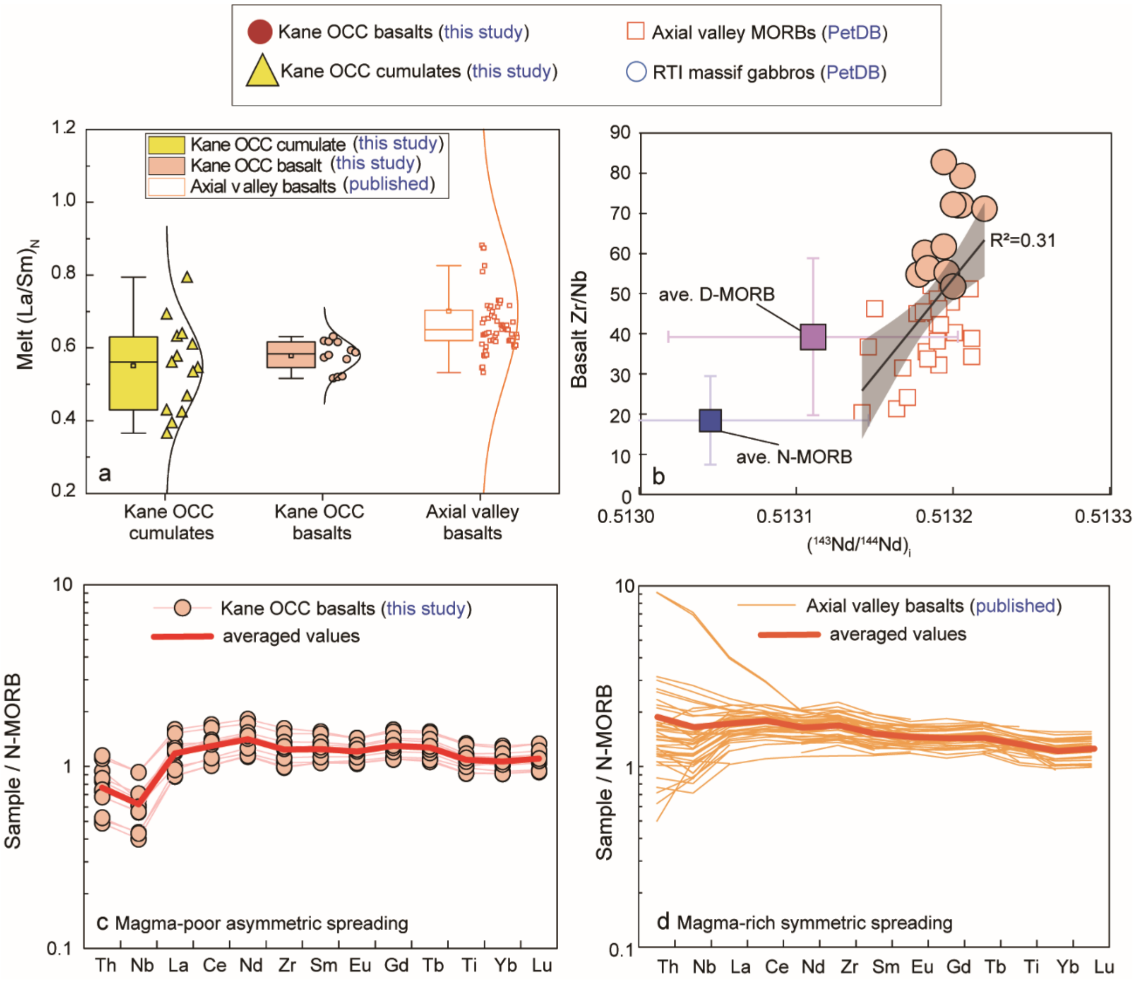

Fig. S5. Geochemical data of basalts and gabbros at MAR 23°N.

a Median $(La/Sm)_N$ ratios for the calculated equilibrium melts of the Kane OCC cumulates, with comparisons with the Kane OCC and axial valley basalts. Details of equilibrium melt composition calculations are given in Supplementary Note 8. **b** Basalt Zr/Nb versus $^{143}Nd/^{144}Nd$. **c-d** N-MORB-normalized trace element patterns of the Kane OCC and axial valley basalts. Sources for published data are provided in Table S6. Averaged compositions of depleted-MORB [D-MORB, $(La/Sm)_N<0.8$] and normally-MORB [N-MORB, $(La/Sm)_N=0.8–1.5$] are calculated using the compiled global MORB dataset from ref. [48] and following classification scheme from ref. [49].

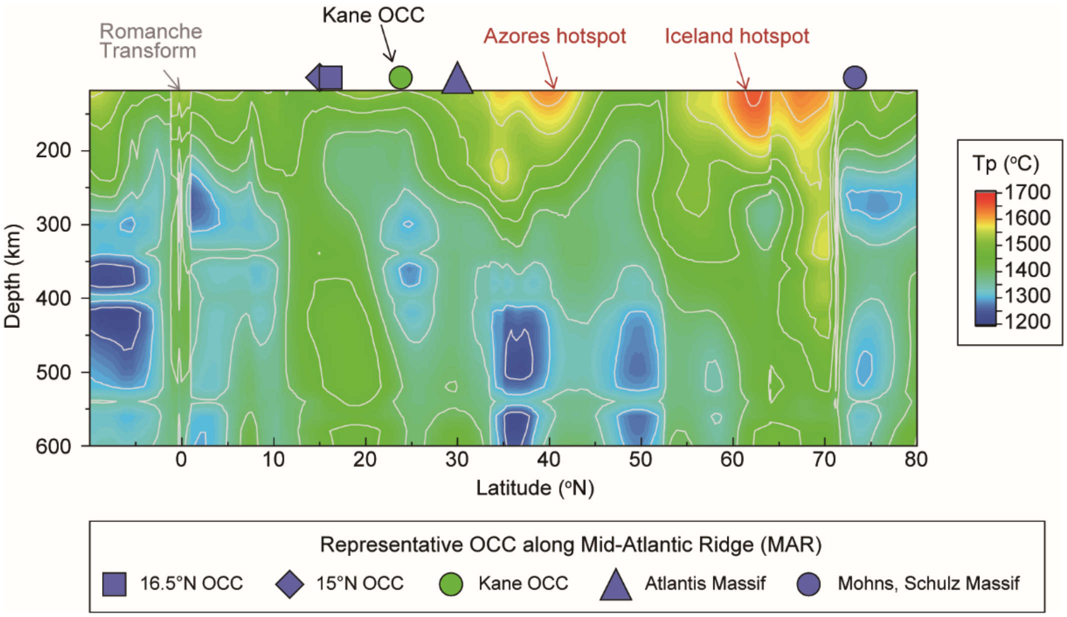

Fig. S6. Distributions of mantle potential temperatures (Tp, °C) at different depths along the Mid-Atlantic Ridge (MAR).

The Tp values were seismically-inferred and derived from Bao et al. [50] (SEMUCB model). The locations of various oceanic core complexes (OCC) in MAR and the two hotspots (Azore and Iceland) are also plotted.

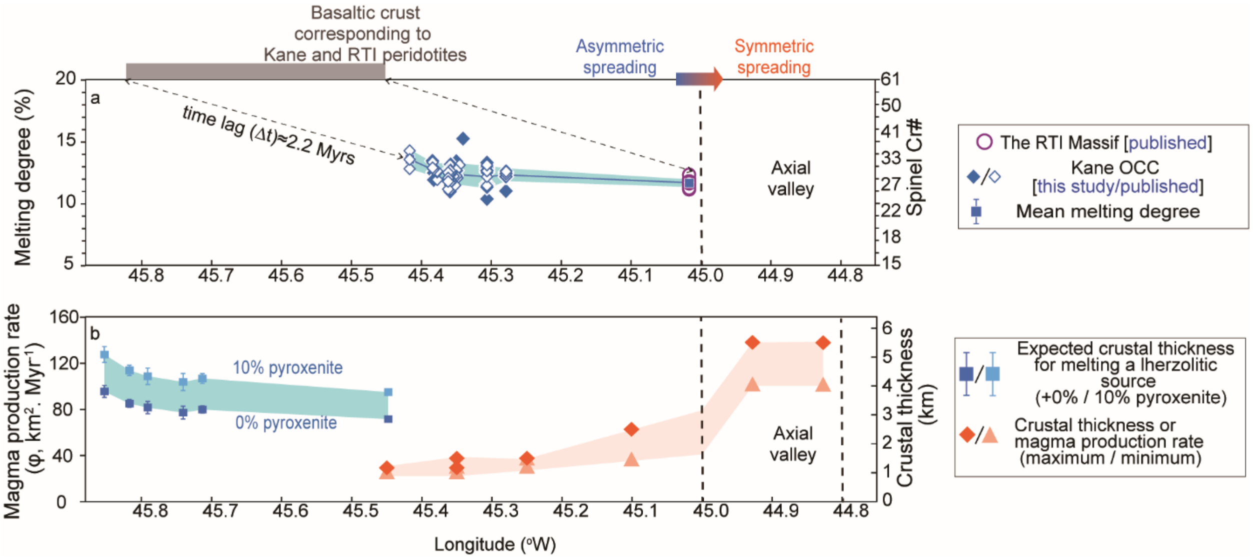

Fig. S7. Cross-axis variations in peridotite melting degrees and crustal thickness, compared with the predictions by Melt-PX simulation.

a Spatial variations of peridotite spinel Cr#, melting degree. **b** Inferred crustal thickness for melting of fertile DMM-like source. The reconstructed initial igneous crustal thickness and the estimated peridotite melting extents for the Kane OCC and RTI Massif are compared with the Melt-PX model predictions.

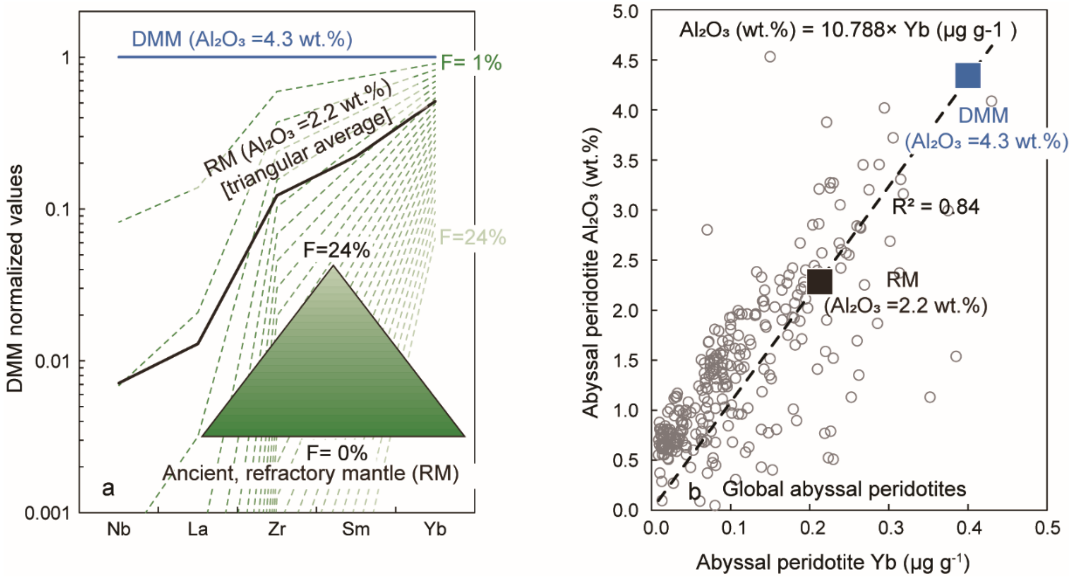

Fig. S8. Reconstruction of the bulk compositions of ancient refractory mantle (RM).

a Illustration of the reconstruction of the Nb, Zr, La, Sm, and Yb compositions of RM. Following the strategy of ref. [19], the bulk compositions (Nb, La, Zr, Sm, Yb) of RM were reconstructed as a weighted sum of single melting intervals (1% melting) from a DMM-like mantle residual in the triangular melting region. During low-pressure spinel-facies melting below ocean ridges, Al_2O_3 and Yb demonstrate similar incompatibility, as evidenced by their strong correlation in residual abyssal peridotites [21], i.e., Al_2O_3 (wt.%)=10.788×Yb(μg g^{-1}), R^2=0.84. **b** Correlation between Al_2O_3 and Yb contents for global abyssal peridotites. Utilizing this relationship, the Yb contents in RM and DMM yielded Al_2O_3 abundances of 2.2 wt.% and 4.3 wt.%, respectively; the latter is consistent with the recommended DMM value [22], confirming the reliability of this estimation. Accordingly, the RM component contains 0.0015 μg g^{-1} Nb, 0.003 μg g^{-1} La, 0.975 μg g^{-1} Zr, 0.060 μg g^{-1} Sm, 0.205 μg g^{-1} Yb, and 2.2 wt.% Al_2O_3, indicating a fertility similar to 7 to 8% melting residues of DMM.

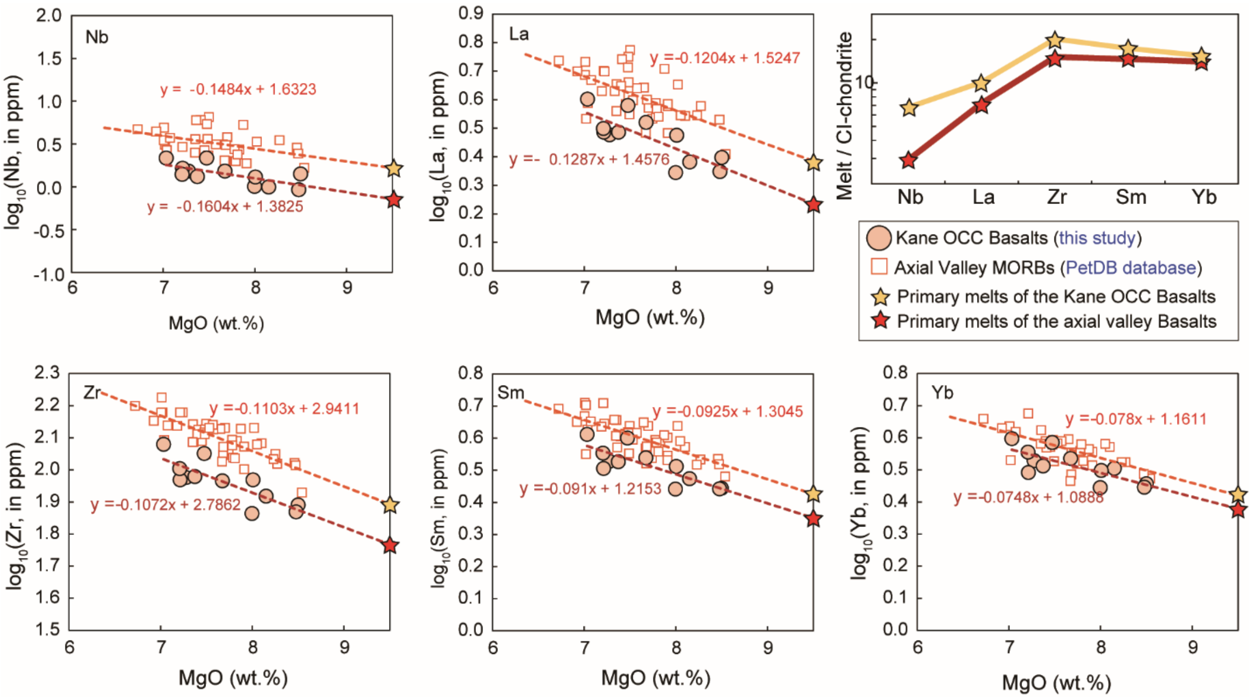

Fig. S9. Reconstruction of the primary melt compositions of the Kane OCC and axial valley basalts.

The published data are compiled in Table S6. This reconstruction was performed following the approach similar to that detailed in ref. [23]. During MORB fractional crystallization, MgO concentration in residual melts is generally linearly correlated with the extent of fractional crystallization and a MgO content of 9.5 wt.% corresponds to a primary melt composition with close to zero degree of fractional crystallization [23]. Similar to the method described in ref. [23], the primary melt trace element (Nb, La, Zr, Sm, Yb) compositions at MgO of 9.5 wt.% were constrained based on the linear correlations between MgO contents and the log [M], where [M] is the content of trace element M in MORB. The primary melt compositions of the Kane OCC and axial valley MORB are given in Table S7.

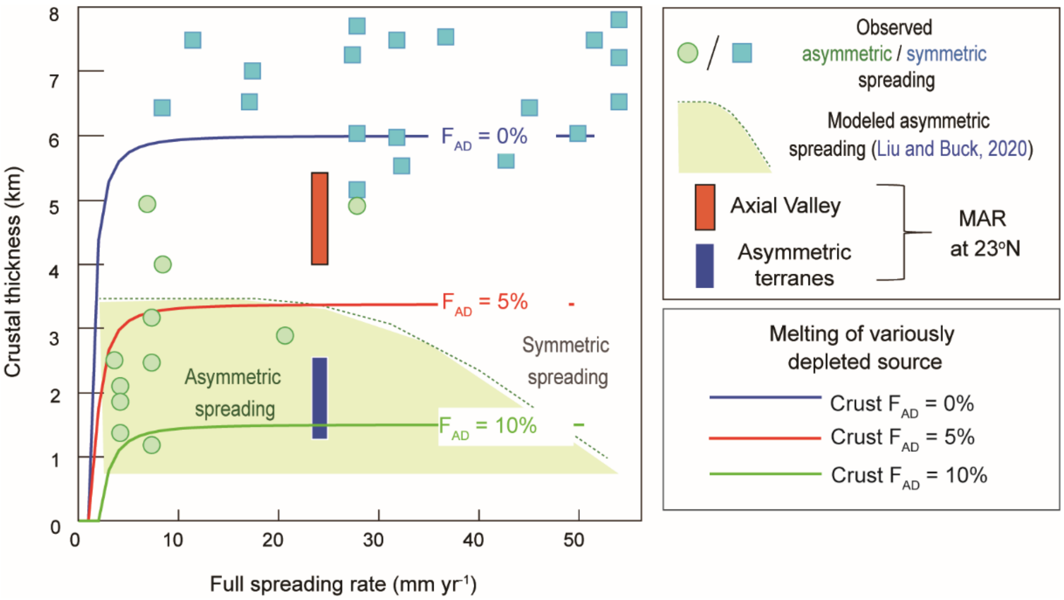

Fig. S10. Possible effect of source depletion on the spreading modes at slow-spreading ridges.

The different spreading modes at various crustal thicknesses and spreading rates are based on the numerical simulations and the compiled global ocean ridge data after ref. [51]. These results suggest that the asymmetric spreading occurs when the full spreading rate is <50 mm yr^{-1} and crustal thickness is <3.5 km. A simplified forward mantle melting models is performed applying the approaches similar to those outlined in Supplementary Note 8, except for applying varying full spreading rates (U_0) and source depletion (F_{AD}) for the former.

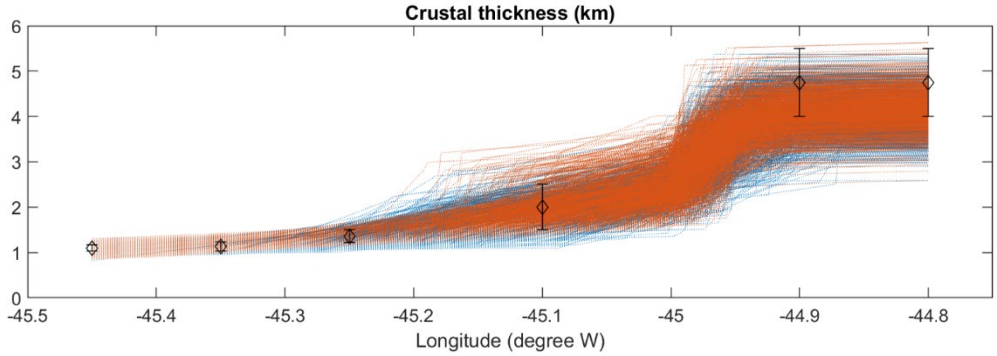

Fig. S11. Modeled crustal thickness by melting of mantle with various prior melt depletion (F_{AD}).

Blue and orange curves represent the results of Model 1 [constant final melting depth (19.5 km) and maximum melting degree (13.5%)] and Model 2 [changing final melting depth (15 to 24 km) and maximum melting degree (12% to 15%)], respectively.

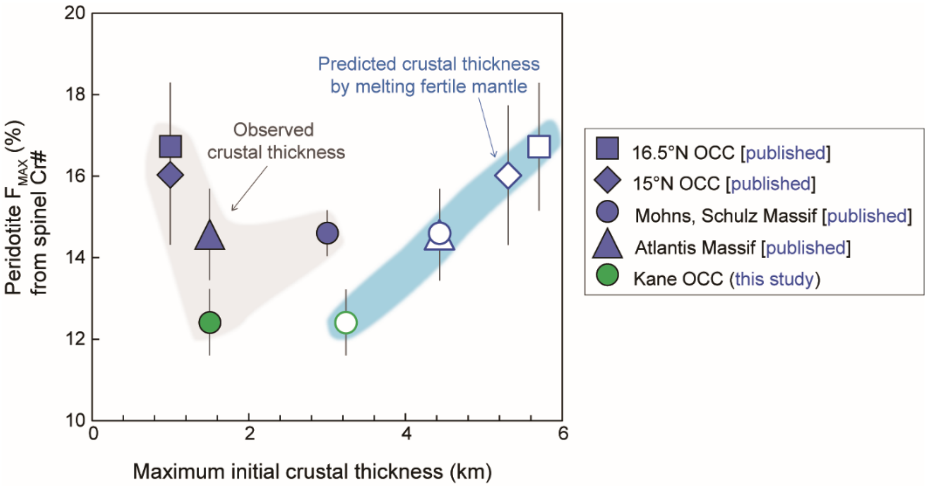

Fig. S12. Summary of peridotite melting degree (F%) and maximum initial crustal thickness (Hc, km) for various Mid-Atlantic Ridge oceanic core complexes.

Correlation of peridotite melting degree with maximum initial crustal thickness (km). Four other OCC from Mid-Atlantic Ridge (MAR) have been examined. These OCC have not been well investigated for high-resolution seismic imaging. Here, we inferred the initial crustal thickness of these OCC based on the proportions and sizes of gabbro intrusions on the footwall. Previous numerical simulation suggests a positive correlation between the proportion of gabbros exposed on the footwall of detachment fault and magma supply [52]. The 14-17ºN OCC consist of peridotites with gabbro intrusions of several to less than one hundred meters in thickness[53], implying a lower initial crustal thickness than the Kane OCC (1.0 to 1.5 km) and leading to a maximum estimate of initial crustal thickness of 1 km for the 14-17ºN OCC. The sizes and proportions of gabbro intrusions for the Atlantis Massif OCC are comparable to those for the Kane OCC, implying a similar initial crustal thickness for them (1.0 to 1.5 km). The crustal thickness for the Schulz Massif OCC from the Mohns Ridge was inferred from deep electrical imaging, i.e., 2.5 to 3.0 km (ref. [29]). The predicted crustal thickness by melting of a fertile mantle was modeled using MeltPX simulation and assuming 0% pyroxenite in source. Data sources: 16.5°N OCC [ref. [54]], 15°N OCC [ref. [43]], Mohns[ref. [55]], Atlantis Massif [ref. [56]].

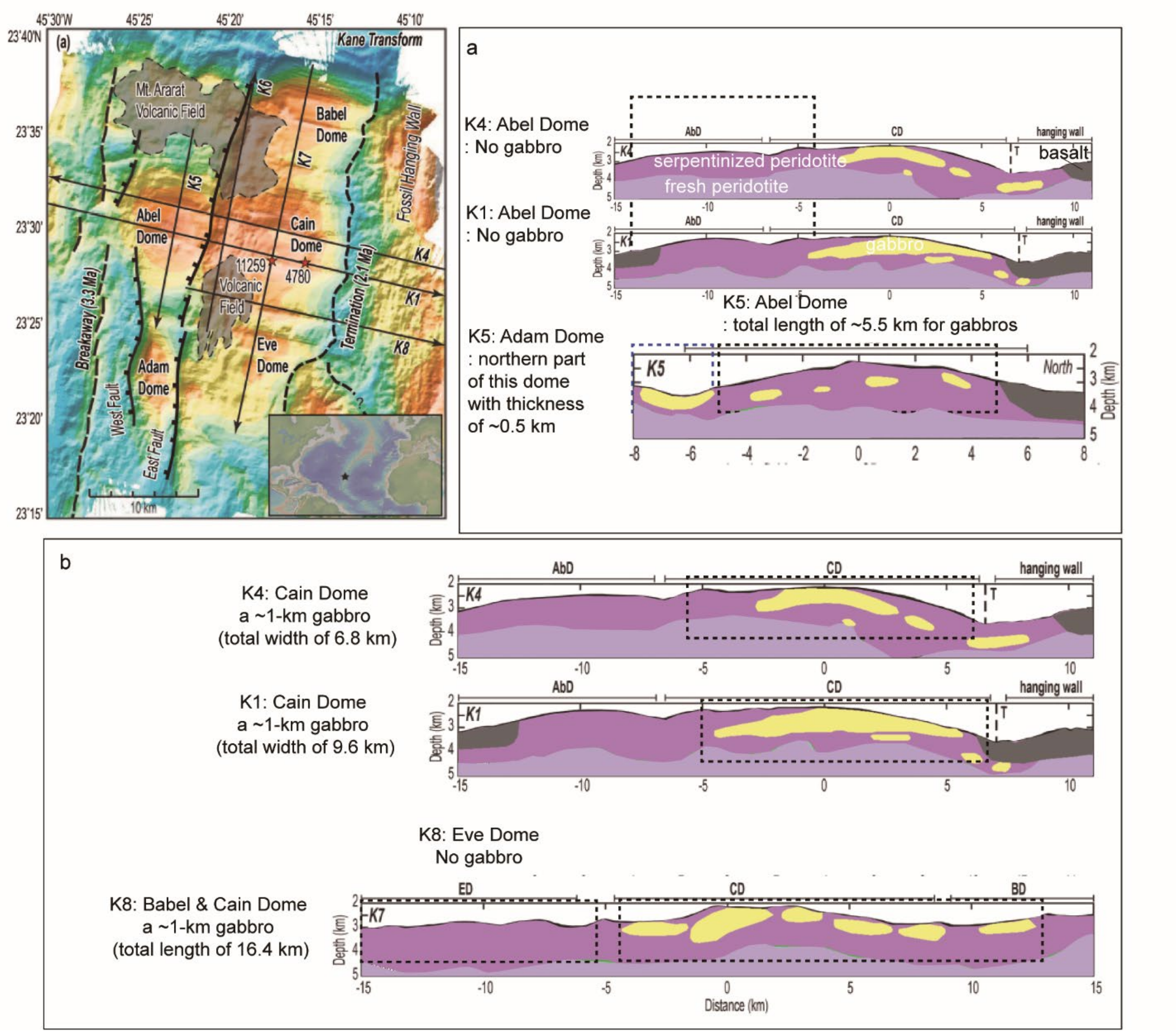

Fig. S13. Interpretations of lithological distributions within the Kane OCC from ref. [4].

a Abel and Adam Dome. Abel Dome: Three profiles (K1, K4, and K5) provide internal architecture of Abel Dome. No gabbro intrusions are detected along profiles K1 and K4 but gabbros with north-to-south total length of 5.5 km are found along profile K5. Though not directly detected along profiles K1 and K4, there remains possibilities that small gabbros with west-to-east length of <0.3 km might exist. We assume these gabbros could have total width value as large as ~5.5 km (their total length) but as small as 0.3 km (similar to the spatial resolution of seismic approach). Adam Dome: Profile K5 reveals that the northern part of the Adam Dome gabbro intrusion has a thickness of ~0.5 km, providing a minimum estimate. Based on seafloor sampling, the Adam Dome gabbro was inferred to have thickness of 0.5 to 1.0 km[2].

b Babel, Cain, and Eve domes. Babel and Cain Domes: Several large gabbros were detected, with total width of 6.8 to 9.6 km, total length of 16.4 km and thickness of 1 km. Eve Dome: no detectable gabbros were found. However, the seafloor sampling implies ~50% basement rocks of the Eve Dome could be gabbros[2]. The thickness of gabbro intrusions below the Eve Dome is inferred to be similar to the Adam Dome (0.5-1.0 km).

Captions for table S1 to S8. Supplementary data including the original geochemical data, compiled literature data, geophysical reconstruction, and geochemical modeling.

Table S1. Whole-rock major and trace elements of Kane OCC peridotites and basalts.

Table S2. Mineral major and trace elements of Kane OCC peridotites.

Table S3. Highly siderophile elements (HSE) and Os isotopes of Kane OCC peridotites.

Table S4. Sr-Nd isotopes of cumulates and basalts from MAR at 23°N.

Table S5. Compiled MORB data for Mid-Atlantic Ridge at 23°N.

Table S6. Geophysical reconstruction of initial igneous crustal thickness of the Kane oceanic core complex (OCC).

Table S7. Geochemical modeling of partial melting of depleted mid-ocean ridge basalt mantle and ancient refractory mantle.

Table S8. Calculated equilibrium melt La/Sm ratios for the Kane OCC cumulates.

Supplementary codes for reproducing the plots in Fig. 6 and Figs. S10 and S11.

Modeling_C++: C++ code for modeling the mantle melting and crustal production at Kane Transform valley and a readme file "Kane_code_readme.docx".

Plot_MATLAB: MATLAB code for visualizing the modeled results.

References:

1. Karson JA, Dick HJB. Tectonics of ridge-transform intersections at the Kane fracture zone. *Mar Geophys Res* 1983; **6**: 51–98.
2. Dick HJB, Tivey MA, Tucholke BE. Plutonic foundation of a slow-spreading ridge segment: Oceanic core complex at Kane Megamullion, 23°30'N, 45°20'W. *Geochem Geophys Geosyst* 2008; **9**: Q05014.
3. Xu M, Canales JP, Tucholke BE et al. Heterogeneous seismic velocity structure of the upper lithosphere at Kane oceanic core complex, Mid-Atlantic Ridge. *Geochem Geophys Geosyst* 2009; **10**: Q10001.

4. Xu M, Zhao X, Canales JP. Structural variability within the Kane oceanic core complex from full waveform inversion and reverse time migration of streamer data. *Geophys Res Lett* 2020; **47**: e2020GL087405.

5. Canales JP, Collins JA, Escartín J et al. Seismic structure across the rift valley of the Mid-Atlantic Ridge at 23º20' (MARK area): Implications for crustal accretion processes at slow spreading ridges. *J Geophys Res* 2000; **105**: 28411–28425.

6. Mével C, Cannat M, Gente P et al. Emplacement of deep crustal and mantle rocks on the west median valley wall of the MARK area (MAR, 23°N). *Tectonophysics* 1991; **190**: 31–53.

7. Brown JR, Karson JA. Variations in axial processes on the Mid-Atlantic Ridge: The median valley of the MARK area. *Mar Geophys Res* 1988; **10**: 109–138

8. Lissenberg CJ, Rioux M, Macleod CJ et al. Crystallization depth beneath an oceanic detachment fault (ODP Hole 923A, Mid-Atlantic Ridge). *Geochem Geophys Geosyst* 2016; **17**: 162–180.

9. Auzende J-M, Cannat M, Gente P et al. Observation of sections of oceanic crust and mantle cropping out on the southern wall of Kane FZ (N. Atlantic). *Terra Nova* 1994; **6**: 143–148.

10. Dick HJB, Lissenberg CJ, Warren JM. Mantle melting, melt transport, and delivery beneath a slow-spreading ridge: The paleo-MAR from 23°15′N to 23°45′N. *J Petrol* 2010; **51**: 425–467.

11. Niu Y. Bulk-rock major and trace element compositions of abyssal peridotites: Implications for mantle melting, melt Extraction and post-melting processes beneath mid-ocean ridges. *J Petrol* 2004; **45**: 2423–2458.

12. Liu J, Riches AJV, Pearson DG et al. Age and evolution of the deep continental root beneath the central Rae craton, northern Canada. *Precambrian Res* 2016; **272**: 168–184.

13. Luguet A, Lorand J-P, Seyler M. Sulfide petrology and highly siderophile element geochemistry of abyssal peridotites: a coupled study of samples from the Kane Fracture Zone (45 °W 23°20′N, MARK area, Atlantic Ocean). *Geochim Cosmochim Acta* 2003; **67**: 1553–1570.

14. Day JMD, Walker RJ, Warren JM. ^{186}Os–^{187}Os and highly siderophile element abundance systematics of the mantle revealed by abyssal peridotites and Os-rich alloys. *Geochim Cosmochim Acta* 2017; **200**: 232–254.

15. Liu C-Z, Snow JE, Brügmann G et al. Non-chondritic HSE budget in Earth's upper mantle evidenced by abyssal peridotites from Gakkel ridge (Arctic Ocean). *Earth Planet Sci Lett* 2009; **283**: 122–132.

16. Day JMD, Brown DB. Ancient melt-depletion in fresh to strongly serpentinized Tonga Trench peridotites. *J Petrol* 2021; **62**.

17. Liu C-Z, Snow JE, Hellebrand E et al. Ancient, highly heterogeneous mantle beneath Gakkel ridge, Arctic Ocean. *Nature* 2008; **452**: 311–316.

18. Salters VJM, Mallick S, Hart SR et al. Domains of depleted mantle: New evidence from hafnium and neodymium isotopes. *Geochem Geophys Geosyst* 2011; **12**.

19. Sanfilippo A, Salters VJM, Sokolov SY et al. Ancient refractory asthenosphere revealed by mantle re-melting at the Arctic Mid Atlantic Ridge. *Earth Planet Sci Lett* 2021; **566**: 116981.

20. Byerly BL, Lassiter JC. Isotopically ultradepleted domains in the convecting upper mantle: Implications for MORB petrogenesis. *Geology* 2014; **42**: 203–206.

21. Regelous M, Weinzierl CG, Haase KM. Controls on melting at spreading ridges from correlated abyssal peridotite – mid-ocean ridge basalt compositions. *Earth Planet Sci Lett* 2016; **449**: 1–11.

22. Salters VJM, Stracke A. Composition of the depleted mantle. *Geochem Geophys Geosyst* 2004; **5**: Q05B07.

23. O'Neill HSC, Jenner FE. The global pattern of trace-element distributions in ocean floor basalts. *Nature* 2012; **491**: 698–705.

24. Workman RK, Hart SR. Major and trace element composition of the depleted MORB mantle (DMM). *Earth Planet Sci Lett* 2005; **231**: 53–72.

25. Forsyth DW. Crustal thickness and the average depth and degree of melting in fractional melting models of passive flow beneath mid-ocean ridges. *J Geophys Res* 1993; **98**: 16073–16079.

26. Warren JM. Global variations in abyssal peridotite compositions. *Lithos* 2016; **248–251**: 193–219.

27. Liu B, Liang Y. Importance of permeability and deep channel network on the distribution of melt, fractionation of REE in abyssal peridotites, and U-series disequilibria in basalts beneath mid-ocean ridges: A numerical study using a 2D double-porosity model. *Earth Planet Sci Lett* 2019; **528**: 115788.

28. Key K, Constable S, Liu L et al. Electrical image of passive mantle upwelling beneath the northern East Pacific Rise. *Nature* 2013; **495**: 499–502.

29. Johansen SE, Panzner M, Mittet R et al. Deep electrical imaging of the ultraslow-spreading Mohns Ridge. *Nature* 2019; **567**: 379–383.

30. Johansen SE, Amundsen H, Arntsen B et al. New model for ultraslow-spreading ridges. 2024.

31. Dasgupta R, Hirschmann MM. Melting in the Earth's deep upper mantle caused by carbon dioxide. *Nature* 2006; **440**: 659–662.

32. Sarafian E, Gaetani GA, Hauri EH et al. Experimental constraints on the damp peridotite solidus and oceanic mantle potential temperature. *Science* 2017; **355**: 942–945.

33. Smith PM, Asimow PD. Adiabat_1ph: A new public front-end to the MELTS, pMELTS, and pHMELTS models. *Geochem Geophys Geosyst* 2005; **6**: Q02004.

34. Zha C, Zhang F, Lin J et al. On the relative importance of buoyancy and thickening of aging lithosphere in mantle upwelling and crustal production beneath global mid-ocean ridge system. *J Geophys Res* 2024; **129**: e2023JB028432.

35. Zhang T, Li J, Niu X et al. Highly variable magmatic accretion at the ultraslow-spreading Gakkel Ridge. *Nature* 2024; **633**: 109–113.

36. Liu B, Liang Y. An introduction of Markov chain Monte Carlo method to geochemical inverse problems: Reading melting parameters from REE abundances in abyssal peridotites. *Geochim Cosmochim Acta* 2017; **203**: 216–234.

37. Canales JP. Small-scale structure of the Kane oceanic core complex, Mid-Atlantic Ridge 23°30′N, from waveform tomography of multichannel seismic data. *Geophys Res Lett* 2010; **37**.

38. Zhang W-Q, Liu C-Z, Johan Lissenberg C et al. Post-cumulus control on copper isotopic fractionation during oceanic intra-crustal magmatic differentiation. *Geochim Cosmochim Acta* 2024; **369**: 35–50.

39. Lissenberg CJ, Macleod CJ. A reactive porous flow Control on mid-ocean ridge magmatic evolution. *J Petrol* 2016; **57**: 2195–2220.

40. Lissenberg CJ, MacLeod CJ, Howard KA et al. Pervasive reactive melt migration through fast-spreading lower oceanic crust (Hess Deep, equatorial Pacific Ocean). *Earth Planet Sci Lett* 2013; **361**: 436–447.

41. Sun C, Liang Y. Distribution of REE between clinopyroxene and basaltic melt along a mantle adiabat: effects of major element composition, water, and temperature. *Contrib Mineral Petrol* 2012; **163**: 807–823.

42. Harvey J, Gannoun A, Burton KW et al. Ancient melt extraction from the oceanic upper mantle revealed by Re–Os isotopes in abyssal peridotites from the Mid-Atlantic ridge. *Earth Planet Sci Lett* 2006; **244**: 606–621.

43. Lin Y-Z, Day JMD, Brown DB et al. Evidence for large-scale, long-term highly siderophile element heterogeneities in the Atlantic mantle from Leg 153 and 209 peridotites. *Geochim Cosmochim Acta* 2024; **378**: 300–314.

44. Lassiter JC, Byerly BL, Snow JE et al. Constraints from Os-isotope variations on the origin of Lena Trough abyssal peridotites and implications for the composition and evolution of the depleted upper mantle. *Earth Planet Sci Lett* 2014; **403**: 178–187.

45. Becker H, Horan MF, Walker RJ et al. Highly siderophile element composition of the Earth's primitive upper mantle: Constraints from new data on peridotite massifs and xenoliths. *Geochim Cosmochim Acta* 2006; **70**: 4528–4550.

46. Ghose I, Cannat M, Seyler M. Transform fault effect on mantle melting in the MARK area (Mid-Atlantic Ridge south of the Kane transform). *Geology* 1996; **24**: 1139–1142.

47. Hellebrand E, Snow JE, Dick HJB et al. Coupled major and trace elements as indicators of the extent of melting in mid-ocean-ridge peridotites. *Nature* 2001; **410**: 677–681.

48. Yang AY, Langmuir CH, Cai Y et al. A subduction influence on ocean ridge basalts outside the Pacific subduction shield. *Nat Commun* 2021; **12**: 4757.

49. Gale A, Dalton CA, Langmuir CH et al. The mean composition of ocean ridge basalts. *Geochem Geophys Geosyst* 2013; **14**: 489–518.

50. Bao X, Mittal T, Lithgow-Bertelloni CR. Determining mid-ocean ridge geography from upper mantle temperature. *Earth Planet Sci Lett* 2024; **641**: 118823.

51. Liu Z, Buck WR. Global trends of axial relief and faulting at plate spreading centers imply discrete magmatic events. *J Geophys Res* 2020; **125**: e2020JB019465.

52. Olive J-A, Behn MD, Tucholke BE. The structure of oceanic core complexes controlled by the depth distribution of magma emplacement. *Nat Geosci* 2010; **3**: 491–495.

53. Kelemen P, Kikawa E, Miller D et al. Drilling mantle peridotite along the Mid-Atlantic Ridge from 14° to 16°N. In: Kelemen PB, Kikawa E,

and Miller DJ (ed.). *Proc ODP Init Repts*. Ocean Drilling Program, 2004, 1–139.

54. Urann BM, Dick HJB, Parnell-Turner R et al. Recycled arc mantle recovered from the Mid-Atlantic Ridge. *Nat Commun* 2020; **11**: 3887.

55. Bjerga A, Stubseid HH, Pedersen LER et al. A highly depleted and subduction-modified mantle beneath the slow-spreading Mohns Ridge. *Geochem Geophys Geosyst* 2022; **23**: e2022GC010585.

56. Akizawa N, Früh-Green GL, Tamura A et al. Compositional heterogeneity and melt transport in mantle beneath Mid-Atlantic Ridge constrained by peridotite, dunite, and wehrlite from Atlantis Massif. *Lithos* 2020; **354–355**: 105364.
